# Ti_3_C_2_ MXene-Based Composites for Hydrogen and Ammonia Gas Sensing: A Review

**DOI:** 10.3390/nano16150955

**Published:** 2026-08-03

**Authors:** Adem Sreedhar, Jin-Seo Noh

**Affiliations:** Department of Physics and Semiconductor Science, Gachon University, 1342 Seongnamdaero, Sujeong-gu, Seongnam-si 461-701, Gyeonggi-do, Republic of Korea; ademgu@gachon.ac.kr

**Keywords:** Ti_3_C_2_ MXene, gas sensing, room temperature, flexible, long term

## Abstract

The unique contributions of 2D Ti_3_C_2_ MXenes surface, electrical, and chemical features play a crucial role in determining toxic and flammable gas-sensing behavior. Specifically, its high electrical conductivity (metallic nature), layered nanosheet structure (nanosheets), and surface termination groups (–O, –F, and –OH) collectively contribute to excellent hydrogen (H_2_) and ammonia (NH_3_) gas-sensing behavior. This review systematically explores the impact of pristine and modified Ti_3_C_2_ MXene, including its interfaces with various metals and metal oxides for enhancing H_2_ and NH_3_ detection. Furthermore, the significance of room temperature operation and flexible gas sensing mechanisms is explored. Notably, integration of Ti_3_C_2_ MXene and sulfur nanosheets demonstrates rapid response and recovery times with detection limits at ppt level. Ti_3_C_2_ MXene-based interfaces also exhibit excellent long-term stability under various relative humidity conditions. The selective surface termination groups (–OH and –O) facilitate the formation of hydrogen bonds with NH_3_ molecules for enhancing gas adsorption and sensing selectivity. In addition, the expansion of the interlayer spacing plays a vital role in improving the gas-sensing performance. Partial oxidation of Ti_3_C_2_ MXene into TiO_2_ increases the interlayer distance, promoting faster diffusion of gas molecules and quicker sensor response. Overall, the intrinsic properties of Ti_3_C_2_ MXene and its composites significantly achieve high-performance room-temperature H_2_ and NH_3_ gas-sensing performance.

## 1. Introduction

In recent decades, rapid industrialization has led to a significant increase in release of toxic gases into the atmosphere, which results in air pollution. Interaction and exposure to these toxic gases poses serious risks to human health, particularly by causing respiratory disorders. It is to be noted that H_2_ and NH_3_ gases play a crucial role in advancing a sustainable and clean energy future [1]. To achieve global net-zero emissions targets, H_2_ fuel is increasingly being adopted as a clean energy carrier for fuel cell vehicles and other energy applications [2]. However, the safe production, storage, and transportation of H_2_ fuel remain major challenges due to high flammability. It is necessary to avoid the interaction of H_2_ concentrations above the lower explosive limit of 4% with explosive materials, which makes the rapid detection of H_2_ leaks essential for ensuring operational safety [3]. Similarly, NH_3_ is a toxic gas and its permissible exposure limit is restricted to 25 ppm in 8 h, according to the standards of the Occupational Safety and Health Administration (OSHA) [4,5]. Interaction between metal and metal oxides strengthens gas-sensing behavior [6]. Therefore, the development of highly sensitive, selective, and reliable gas sensors for H_2_ and NH_3_ detection is of considerable importance. Semiconductor metal oxides and their heterostructures have been extensively investigated for gas sensing because of their excellent chemical stability and tunable electronic properties [7]. In particular, transition metal oxides such as ZnO, WO_3_, MoO_3_, TiO_2_, and SnO_2_ (both n- and p-type) have demonstrated promising H_2_ and NH_3_ gas-sensing performance [8,9,10,11,12]. Nevertheless, conventional metal oxide sensors generally require elevated operating temperatures; they exhibit high sensitivity to ambient humidity, which decreases their H_2_ gas-sensing ability. Meanwhile, decrements in the recovery process (desorption) of transition metal oxides during the NH_3_ gas-sensing process have been noted. To address these limitations, forms of 2D Ti_3_C_2_ MXene have emerged as promising gas-sensing materials owing to their exceptional metallic conductivity, ultrathin layered structure, large specific surface area with abundant exposed active sites, and surface termination groups (–O, –OH, and –F), which provide favorable adsorption sites for gas molecules [13]. These unique characteristics make 2D Ti_3_C_2_ MXene an attractive material for room temperature H_2_ and NH_3_ gas sensing. Interestingly, Ti_3_C_2_ MXene exhibits excellent H_2_ storage capability when integrated with various metal hydrides for highlighting its potential for hydrogen-related applications [14]. Its layered architecture facilitates rapid gas diffusion and high electrical conductivity (fast charge transport), which results in enhanced sensitivity, rapid response/recovery times, and improved room-temperature gas-sensing performance. Rapid charge transport kinetics also play a crucial role in determining the gas-sensing behavior of Ti_3_C_2_ MXene [15]. The adsorption energy of NH_3_ on Ti_3_C_2_O_2_ has been reported to be 0.42 eV accompanied by a charge transfer rate of 0.18 e [16], which indicates a favorable interaction between NH_3_ molecules and the MXene surface. In detail, as NH_3_ contains a lone electron pair, it acts as an electron donor facilitating electron transfer to Ti_3_C_2_O_2_ and inducing a significant change in its electrical resistance for strengthened gas-sensing response/recovery signals. Interestingly, the surface –OH termination groups on Ti_3_C_2_ MXene promote the formation of strong hydrogen bonds with NH_3_ molecules to modulate the electrical signals [17]. In other words, the metallic or p-type semiconductor behavior in the Ti_3_C_2_ MXene effectively gains electrons from NH_3_ and recombines with holes, which results in increase of resultant resistance [18]. In addition to H_2_ and NH_3_ detection, the intrinsic properties of 2D Ti_3_C_2_ MXene also facilitate remarkable room-temperature NO_2_ gas sensing [19]. Therefore, Ti_3_C_2_ MXene-based composites have attracted increasing attention as promising materials for room-temperature gas-sensing applications.

This review comprehensively summarizes and evaluates the room-temperature NH_3_ and H_2_ gas-sensing performance of various 2D Ti_3_C_2_ MXene-integrated semiconductor nanostructures. Particular emphasis is placed on the remarkable improvement of the limit of detection (LOD), ranging from ppm to ppt levels. In addition, this review discusses the excellent cyclic durability and long-term stability of these composites under harsh humid environmental conditions. The influence of enlarged interlayer spacing on facilitating gas diffusion and increasing the accessibility of active adsorption sites is also highlighted. Moreover, the role of surface termination groups (–O, and –OH) in promoting hydrogen-bond formation and enhancing gas adsorption is critically examined to provide a comprehensive understanding of the improved gas-sensing performance of Ti_3_C_2_ MXene-based materials.

## 2. Importance of Ti_3_C_2_ MXene for Gas Sensing

**Figure 1:**
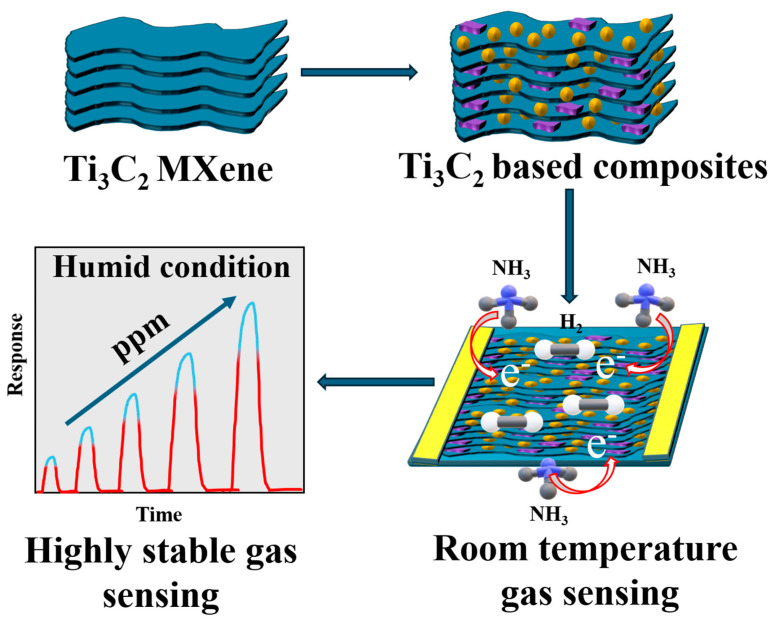
Room temperature NH_3_ and H_2_ gas-sensing performance of various Ti_3_C_2_ MXene nanosheet-integrated nanostructures.

As discussed above, Ti_3_C_2_ MXene exhibits several attractive properties, including a 2D layered structure, high specific surface area, excellent electrical conductivity, and tunable surface terminal groups. These characteristics facilitate active adsorption sites for targeting gas molecules, which result in electrical signal variation and efficient hazardous-gas-sensing performance [20]. In particular, the layered structure promotes gas diffusion by maximizing the number of accessible active sites. The availability of a high surface-area-to-volume ratio provides abundant active edges for gas adsorption, while the surface termination groups serve as chemically active sites for strong interaction with target gas molecules. Furthermore, the high electron density of Ti_3_C_2_ MXene facilitates rapid charge carrier transport during gas adsorption, which leads to enhanced sensing performance. The fabrication of few-layer or monolayer Ti_3_C_2_ MXene nanosheets further increases the exposure of basal-plane active sites, thereby improving gas diffusion and adsorption across the nanosheet surface. Additionally, interlayer expansion achieved through the incorporation of nanoparticles or other 2D nanosheets creates additional diffusion pathways, resulting in faster response and recovery times. Collectively, these unique structural and electronic characteristics of 2D Ti_3_C_2_ MXene establish a highly promising material for room-temperature gas-sensing applications. Figure 1 illustrates the NH_3_ and H_2_ gas-sensing performance ability of Ti_3_C_2_ MXene integrated with various nanostructures.

## 3. Ti_3_C_2_ MXene-Based Composites for H_2_ Gas Sensing

### 3.1. Ti_3_C_2_/Pd Nanostructures

**Figure 2:**
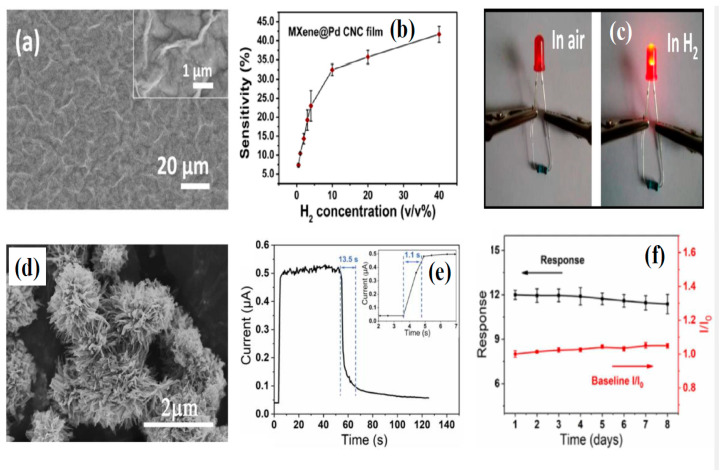
(**a**) Surface morphology of the Ti_3_C_2_ MXene@Pd CNC composite film, (**b**) H_2_ gas-sensing ability of Ti_3_C_2_ MXene@Pd CNC at various H_2_ (*v*/*v*) concentrations, (**c**) demonstration of the practical applicability of Ti_3_C_2_@Pd CNC for H_2_ gas sensing using an LED indicator (Reprinted from Ref. [21], copyright with permission from Elsevier), (**d**) surface morphology of Pd nanoparticles integrated Ti_3_C_2_ MXene-derived sodium titanate nanoribbons, (**e**) rapid response and recovery times of 1.1 s and 13.5 s by the Pd-NTO NRs composite, and (**f**) long-term stable response of the Pd-NTO NRs composite over 8 days (Reprinted from Ref. [23], copyright with permission from Elsevier).

The effective interaction between Ti_3_C_2_ MXene and ultra-small Pd colloidal nanostructures (Pd CNC) (5 ± 1.9 nm) significantly enhances H_2_-sensing performance [21]. in this example, upon H_2_ exposure, the formation of PdH_x_ altered the Pd work function (3.2 eV), facilitating electron transfer from Pd to the Ti_3_C_2_ MXene. Furthermore, the intercalation of Pd CNCs between Ti_3_C_2_ MXene nanosheets increased the interlayer spacing, resulting in a larger effective surface area for gas adsorption. The surface morphology of the Ti_3_C_2_ MXene@Pd CNC film is presented in Figure 2a. Accordingly, the Ti_3_C_2_ MXene@Pd CNC film sensor exhibited stable response and recovery characteristics during H_2_ detection. In this system, Ti_3_C_2_ MXene exhibited n-type semiconducting behavior and effectively accepted electrons from Pd the to Ti_3_C_2_ MXene, which led to a decrease in electrical resistance. The sensor demonstrated response and recovery times of 32 ± 7 s and 161 ± 23 s at a low H_2_ concentration of 4%. In detail, accelerated electron transfer from Pd CNC to Ti_3_C_2_ MXene was observed after insertion of H_2_ under high H_2_ adsorption. Under these conditions, the flexible paper-based Ti_3_C_2_@Pd CNC sensor film achieved a sensitivity of 23.0 ± 4.0% at 4% H_2_, as shown in Figure 2b. In addition, the sensor maintained stable performance under bending angles of 30, 60, 90, and 120°, demonstrating excellent mechanical flexibility. For practical application, an H_2_ gas leak detection system incorporating an alarm circuit and LED indicator was developed. As illustrated in Figure 2c, after exposure to 4% H_2_, the LED was activated, which confirmed the sensor’s capability for real-time leak detection. Overall, the flexible Ti_3_C_2_ MXene@Pd CNC composite demonstrated considerable potential for practical H_2_ gas-sensing applications due to its high sensitivity, mechanical flexibility, and reliable detection performance.

In another study, Doan et al. [22] fabricated a room-temperature H_2_ gas sensor by decorating multilayer Ti_3_C_2_T_x_ MXene with Pd nanoparticles at different loadings (30, 60, and 90 mg) on silicon substrates. The authors demonstrated that both the Ti_3_C_2_T_x_ MXene loading (60 mg) and Pd nanoparticle content significantly influenced the H_2_ gas-sensing performance at 10, 50, and 100 ppm concentrations. The incorporation of Pd nanoparticles enhanced the H_2_ adsorption and promoted intimate interfacial contact with Ti_3_C_2_T_x_ for facilitating the formation of efficient charge transport pathways. Among the investigated samples, the sensor containing 60 mg of Ti_3_C_2_T_x_ MXene with a lower Pd nanoparticle density exhibited highest response of 56% at 100 ppm H_2_. Furthermore, the sensor showed excellent cyclic stability over five consecutive cycles and maintained a strong sensing response after approximately 90 days of storage under ambient conditions at 50 ppm H_2_. The enhanced sensing performance was attributed to the formation of PdH and TiH_2_ during H_2_ exposure, which increased the electrical resistance of the Pd–Ti_3_C_2_T_x_ composite. Overall, the optimized combination of Ti_3_C_2_T_x_ MXene loading and Pd nanoparticle content resulted in a stable, sensitive, and reliable room-temperature H_2_ gas sensor.

Similarly, Wu et al. [23] combined Ti_3_C_2_ MXene-derived sodium titanate nanoribbons (NTO NRs) and nanotubes (NTO NTs) with Pd nanoparticles (3.5 nm) for room temperature H_2_ gas sensing. Notably, the abundant oxygen vacancies in the NTO NRs facilitated rapid H_2_ adsorption and dissociation, resulting in ultrafast H_2_ sensing performance. The surface morphology of the Pd-NTO NRs is shown in Figure 2d. During H_2_ exposure, formation of PdH_x_ resulted in a lower work function than n-type NTO, which created a favorable Schottky junction that promoted electron transfer from PdH_x_ to the NTO, which enhanced the sensing response. Furthermore, the 1D nanoribbon architecture provided a shorter charge diffusion path than the nanotube structure, which enabled more efficient charge transport under reduced scattering at multiple interconnections. XPS studies (O 1s spectrum) of the Pd-NTO NRs showed the presence of –OH and oxygen vacancy peaks, which influenced the gas adsorption process. Accordingly, the Pd-NTO NRs achieved an ultrafast response time of 1.1 s (recovery-13.5 s) with a response of 12.0 toward 1% H_2_, as shown in Figure 2e. The sensor also demonstrated a wide detection range from 0.8 ppm to 10% H_2_. The enhanced sensing mechanism was mainly attributed to the catalytic activity of Pd nanoparticles, which dissociated H_2_ molecules into atomic H. These H atoms spilled over onto the NTO surface, which reacted with adsorbed O_2_^−^ ions and released the electrons, thereby increasing the sensor current. Meanwhile, the formation of O–H and Ti–H surface bonds further contributed to the enhanced electrical response. Moreover, the Pd-NTO NR sensor exhibited excellent long-term stability, maintaining its H_2_ sensing performance even after 8 days, as illustrated Figure 2f. Overall, the synergistic interaction between Pd nanoparticles and Ti_3_C_2_ MXene-derived NTO nanoribbons significantly enhanced the ultrafast and highly sensitive detection of H_2_ gas.

In addition to the above incorporation of Pd nanostructures, Wang et al. [24] modified the Ti_3_C_2_T_x_ MXene surface by integrating bimetallic=Pt nanoparticles and Pd-Pt alloy for the elevation of room-temperature H_2_ gas sensing. The Pd=Pt/Ti_3_C_2_T_x_ exhibited a response time of 6 s and recovery time of 8 s with a remarkable response of 24.6% towards 200 ppm H_2_. Notably, the sensor demonstrated excellent micro-leak detection capability with LOD as low as 1 ppm at an ultrafast response time of 1 s. This response time was slightly faster than that of reported Pd-NTO NRs sensor (1.1 s) at 1% H_2_ [23]. The enhanced sensing performance was attributed to the modulation in the Schottky junction formed between the Pd=Pt nanoparticles and Ti_3_C_2_T_x_ MXene. In air, adsorbed oxygen molecules extracted electrons from the MXene surface, leading to the formation of an electron depletion layer. Upon exposure to H_2_, the catalytic dissociation of H_2_ on the Pd–Pt nanoparticles promoted electron transfer back to the Ti_3_C_2_T_x_ surface, altering the depletion layer and decreasing the sensor resistance. Among the two metals, Pd exhibited a higher H_2_ adsorption capability than Pt, playing a dominant role in improving the sensing performance of the bimetallic composite. The Pd=Pt/Ti_3_C_2_T_x_ sensor maintained stable sensing characteristics over 10 consecutive operating cycles, demonstrating excellent repeatability. Overall, the incorporation of bimetallic Pd=Pt nanoparticles significantly improved the room-temperature H_2_-sensing performance, enabling highly sensitive detection of H_2_ micro-leaks down to 1 ppm.

### 3.2. Ti_3_C_2_ MXene Completely Derived C/TiO_2_

Ti_3_C_2_ MXene can be completely converted into C/TiO_2_ at certain elevated temperatures, offering a promising route for developing environmental safety devices [25]. In this context, Ti_3_C_2_ MXene-derived C/TiO_2_ composites prepared at 400 and 500 °C were systematically investigated for H_2_ gas sensing at room temperature as well as at elevated temperatures (100, 150, 200, 250 and 300 °C) [26]. Decrease in corban and fluorine content along with an increase in oxygen content (TiO_2_ formation) was observed following increased calcination temperatures (400 and 500 °C). Here, oxygen was diffused into the Ti_3_C_2_ MXene, confirming the partial oxidation of Ti_3_C_2_ MXene into TiO_2_. The O 1s spectra revealed oxygen vacancies at 533.7 eV. In detail, H_2_ interaction with lattice oxygen of C/TiO_2_ transformed into oxygen vacancy, which released electrons into the conduction band and lowered the resistance. Among the prepared samples, the C/TiO_2_ composite calcined at 500 °C exhibited the best sensing performance, delivering a response of 92.6% toward 40,000 ppm H_2_ at 300 °C, with response and recovery times of 45 and 20 s, respectively. Remarkably, the same sensor also demonstrated excellent room-temperature performance by achieving a response of 62.2%, with response and recovery times of 13 and 42 s, respectively. The improved sensing performance at elevated temperatures was mainly attributed to enhanced surface reaction kinetics, reduced interference from humidity and volatile impurities, and the effective removal of reaction by-products. Overall, the controlled thermal transformation of Ti_3_C_2_ MXene into a C/TiO_2_ composite significantly improved the stability of H_2_ sensing performance over a wide range of operating temperature.

### 3.3. Ti_3_C_2_ MXene/Organic Frameworks

In addition to noble metal-functionalized Ti_3_C_2_ MXene sensors, Nallamalla et al. [27] developed a flexible room-temperature H_2_ gas sensor by integrating a covalent organic framework (DAAQ-COF) with Ti_3_C_2_ MXene on OHP sheet substrate. The resulting DAAQ-COF/Ti_3_C_2_ MXene composite exhibited enhanced structural, chemical, and morphological stability owing to strong interfacial electronic modulation at the COF/MXene interface. Notably, optimizing the Ti_3_C_2_ MXene content to 50 wt% effectively regulated the interfacial charge transfer and H_2_ adsorption behavior, leading to superior sensing performance. The adsorption strength (chemisorption) and heterointerface phenomena significantly contributed to improved room-temperature H_2_ gas sensing. In this process, the flexible sensor achieved a LOD of 1 ppm with response and recovery times of 29 and 18 s, respectively, at room temperature. The response and recovery times were increased at an elevated gas-sensing temperature of 150° (74/88 s) and bending angle of 97.45° (85/87 s). Overall, the integration of DAAQ-COF with Ti_3_C_2_ MXene provided an effective strategy for developing flexible, highly sensitive H_2_ sensors, capable of detecting H_2_ at ppm-level concentrations while maintaining good operational stability under thermal and mechanical stress.

### 3.4. Ion-Beam Irradiated Ti_3_C_2_ MXene

**Figure 3:**
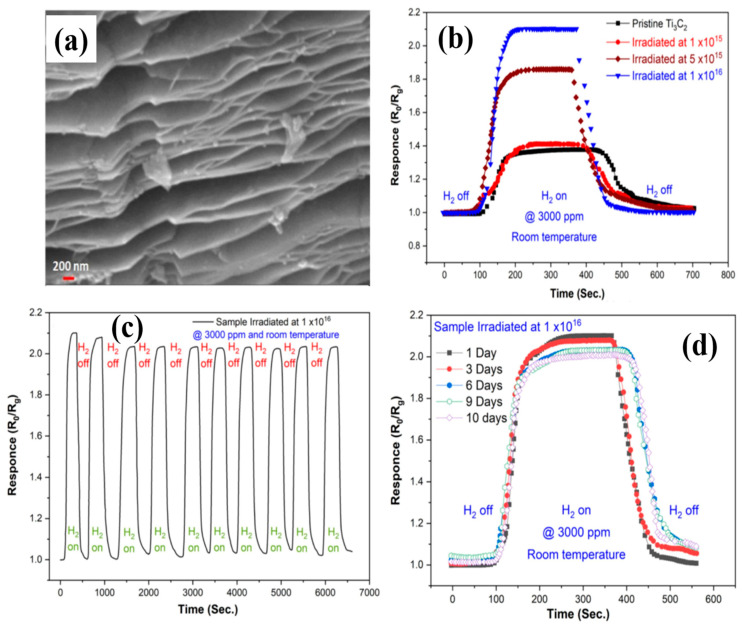
(**a**) Surface morphology of Ti_3_C_2_ MXene nanosheets irradiated with an N^+^ ion-beam at a fluence of 1 × 10^16^ ions cm^−2^, (**b**) response and recovery characteristics of Ti_3_C_2_ MXene irradiated under different N^+^ ion-beam energies. (**c**) room temperature cyclic stability over 10 sensing cycles for Ti_3_C_2_ MXene irradiated at a fluence of 1 × 10^16^ ions cm^−2^, (**d**) remarkable long-term stability over 1 to 10 days for Ti_3_C_2_ MXene irradiated at a fluence of 1 × 10^16^ ions cm^−2^ (Reprinted from Ref. [30], copyright with permission from Elsevier).

Previously, ion-beam irradiation has been demonstrated as an effective approach for modifying the physical properties of semiconductor thin films, such as Al and Cu-doped ZnO systems [28,29]. Following this strategy, low-energy N^+^ ion-beam irradiation with fluences of 1 × 10^15^, 5 × 10^15^, and 1 × 10^16^ ions cm^−2^ was employed to modify Ti_3_C_2_ MXene nanosheets to achieve room-temperature H_2_ gas sensing [30]. The ion-beam treatment induced subtle structural modifications in the Ti_3_C_2_ MXene nanosheets, including reduced crystallite size, improved nanosheet ordering, and increased interlayer d-spacing. The surface morphology of Ti_3_C_2_ MXene irradiated with 1 × 10^16^ ions cm^−2^ is shown in Figure 3a. Among the different irradiation conditions, the Ti_3_C_2_ MXene treated at ion fluence of 1 × 10^16^ ions cm^−2^ exhibited higher H_2_ sensing response of 2.1 toward 3000 ppm H_2_, with response and recovery times of 98 and 109 s, respectively (Figure 3b). In comparison, pure Ti_3_C_2_ MXene displayed a lower response of 1.37 and significantly longer response and recovery times of 170 and 244 s, respectively. Furthermore, the ion-beam-modified Ti_3_C_2_ MXene demonstrated excellent cyclic stability over 10 sensing cycles and retained 94% of its initial response after 10 days (Figure 3c,d). After the interaction of Ti_3_C_2_ MXene with H_2_, the electrons effectively transferred from H_2_ to the Ti_3_C_2_ MXene, which increased the electron density in the sensor. Overall, ion-beam-induced structural engineering effectively optimized the surface and electronic properties of Ti_3_C_2_ MXene, resulting in improved room-temperature H_2_-sensing performance with enhanced stability.

Overall, Ti_3_C_2_ MXene-based sensors have exhibited enhanced H_2_ gas-sensing performance through structural modification and hybridization. Integration with Pd nanoparticles, Pd-Pt alloys, MXene-derived sodium titanate nanoribbons, COF frameworks, and ion-beam treatment features significantly improved their sensitivity, response/recovery times, stability, and LOD. Specifically, Pd facilitated H_2_ dissociation and electron transfer, while engineered interfaces enhanced charge transportation and adsorption. Flexible Ti_3_C_2_/Pd composites detected 4% H_2_ with stable operation under bending conditions. Interestingly, bi-metallic Pd-Pt/Ti_3_C_2_ composite achieved 1 ppm LOD, and DAAQ-COF/Ti_3_C_2_ also sensed 1 ppm H_2_ at room temperature. On the other hand, Ti_3_C_2_ MXene-derived C/TiO_2_ and ion-beam-modified MXene further demonstrated improvement in H_2_-sensing performance and durability. The progress in Ti_3_C_2_T_x_ MXene-based composites for room temperature H_2_ gas sensing is presented in Table 1.

## 4. Ti_3_C_2_ MXene-Based Composites for NH_3_ Gas Sensing

In addition to H_2_ gas sensing, Ti_3_C_2_-based composites have been used for NH_3_ gas sensing at room temperature. Specifically, ultrafast response and recovery processes were achieved in the developed composites. The dominant performance of pure Ti_3_C_2_ and Ti_3_C_2_-based composites is described below.

### 4.1. Pure Ti_3_C_2_ MXene

The room-temperature NH_3_ gas-sensing performance of Ti_3_C_2_T_x_ MXene films with thickness ranging from 2.5 (0.01 mg·mL^−1^) to 15 nm (2.56 mg·mL^−1^) was investigated [31]. The authors reported that thinner Ti_3_C_2_T_x_ MXene films (2.5 nm) exhibited higher sensitivity toward NH_3_ molecules than thicker films. The 2.5 nm Ti_3_C_2_T_x_ MXene films displayed semiconducting behavior due to the presence of surface termination groups, which induced dipole polarization. Thus, a remarkable difference in NH_3_ sensing performance between the thinner and thicker films was observed. Notably, the 2.5 nm film exhibited a response of 1.22% toward 1 ppm NH_3_, whereas the thicker films showed no detectable response at this concentration. Furthermore, the thinnest film achieved a maximum response of 9.4% at 100 ppm, with excellent cyclic stability over five sensing cycles. As the thickness increased from 2.5 to 15 nm, the NH_3_ gas sensing rapidly decreased from 9.4 to 0.35%, respectively. These findings demonstrate that precise control of Ti_3_C_2_T_x_ MXene film thickness plays a crucial role in enhancing room-temperature NH_3_ gas-sensing performance.

Interestingly, the strategic combination of MXene and its parent MAX phase (Ti_3_C_2_T_x_/Ti_3_AlC_2_) enabled efficient room-temperature NH_3_ gas sensing [32]. The lower work function of Ti_3_AlC_2_ compared to Ti_3_C_2_T_x_/Ti_3_AlC_2_ composite confirmed the formation of a planar heterojunction formation at the Ti_3_C_2_T_x_/Ti_3_AlC_2_ interface. Furthermore, the Ti_3_C_2_T_x_ MXene surface contained higher concentrations of –OH and –O termination groups than –F groups, which provided abundant active sites for NH_3_ adsorption. According to the O1 s XPS spectrum, the binding energies of adsorbed oxygen species, Ti–O–Ti, Ti–OH, and C–OH were located at 529.51, 530.22, 531.95, and 532.32 eV, respectively. Upon exposure to NH_3_, these oxygen-containing functional groups facilitated electron transfer to the Ti_3_C_2_T_x_/Ti_3_AlC_2_ composite, resulting in an increase in sensor resistance. Moreover, the formation of a Schottky barrier at the heterointerface promoted efficient charge carrier separation and enhanced interaction between the sensing surface and NH_3_ molecules, which improved sensing performance. In this context, the Ti_3_C_2_T_x_/Ti_3_AlC_2_ composite exhibited a response of 1.2% at a LOD of 50 ppb. At 200 ppm, the sensor achieved a high response of 11.0% along with rapid response and recovery times of 12 and 60 s, respectively. Furthermore, the responses towards CO_2_ and ethanol were lower at about 1.5 and 2.2% compared to NH_3_ (8.3%) at 100 ppm, which confirmed the high selectivity of the Ti_3_C_2_T_x_/Ti_3_AlC_2_ heterostructure towards NH_3_. In addition, the synergistic interaction between Ti_3_C_2_T_x_ and Ti_3_AlC_2_ contributed to excellent long-term stability with negligible degradation after 120 days. Furthermore, DFT calculations demonstrated that the –OH-terminated Ti_3_C_2_(OH)_2_/Ti_3_AlC_2_ heterostructure had higher NH_3_ adsorption capability compared with pristine Ti_3_C_2_O_2_ and Ti_3_C_2_F_2_, owing to stronger binding interactions. Among these, Ti_3_C_2_(OH)_2_/Ti_3_AlC_2_ exhibited higher NH_3_ adsorption energy (−0.950 eV) than Ti_3_C_2_O_2_ (−0.705 eV) and Ti_3_C_2_F_2_ (−0.467 eV), which indicated superior adsorption performance and potential for improving NH_3_ gas-sensing performance. Overall, the combined effects of (i) Ti_3_C_2_T_x_/Ti_3_AlC_2_ heterostructure, (ii) abundant –OH and –O surface termination groups, and (iii) Schottky barrier formation enhanced the room-temperature NH_3_-sensing performance.

Recently, Mirzaei et al. [33] explored the NH_3_-sensing performance of pure Ti_3_C_2_T_x_ MXene (24 h etched) under self-heating conditions between 1 to 6 V applied voltages. With regard to NH_3_, acetone, methanol, ethanol, benzene, toluene, and H_2_S sensing, Ti_3_C_2_T_x_ MXene was highly selective towards 100 ppm NH_3_, with a response of 0.67% at 25 °C and 4 V. Partial oxidation of Ti_3_C_2_T_x_ MXene into TiO_2_ revealed –O surface termination groups. According to the XPS O 1 s spectra, lattice oxygen from Ti–O and adsorbed –OH species at 530.1 and 532.2 eV enhanced the NH_3_ adsorption. The presence of –OH, –O, and –F accelerated the NH_3_ adsorption and electron transfer and improved NH_3_ selectivity. The response to higher humidity conditions (30 to 90%) was not much altered compared with 0% (dry air) conditions. In detail, the response at 90% relative humidity was 0.502% (22% reduction) compared with 0% humidity (0.67%) at 100 ppm NH_3_, demonstrating tolerance under harsh humid conditions. The response was unaltered at 0.613%, with no significant drift even after six cycles. Also, the sensor was sustained through 500 bending and 1000 tilting cycles, with no structural changes after mechanical testing.

### 4.2. Metal-Ions Intercalated Ti_3_C_2_ MXene

Kim et al. [34] strategically intercalated the K^+^, Na^+^, Ca^2+^, and Mg^2+^ cations between Ti_3_C_2_T_x_ MXene nanosheets for effective room-temperature NH_3_ gas sensing from 10 to 100 ppm. The Na^+^ ions-intercalated Ti_3_C_2_T_x_ MXene showed a better response of about 0.36% at 100 ppm compared with the other metal cation counterparts. A signal-to-noise ratio of 531 was achieved for Na^+^-Ti_3_C_2_T_x_ MXene, which was higher than K^+^-Ti_3_C_2_T_x_ (192), Mg^2+^-Ti_3_C_2_T_x_ (333), and Ca^2+^-Ti_3_C_2_T_x_ (371) MXenes. The intercalation process facilitated preparation of transparent and highly conductive films for attaining stable electrical pathways during the gas-sensing process. This strategy did not lead to oxidation and TiO_2_ formation, thereby preserving the pure chemical structure of Ti_3_C_2_T_x_ MXene. Overall, metal ion intercalation increased the gas-sensing performance of Ti_3_C_2_T_x_ MXene.

### 4.3. Alkalized Ti_3_C_2_ MXene

The electrical properties of Ti_3_C_2_T_x_ MXene can be effectively altered by alkalization [35]. In this context, alkalization of Ti_3_C_2_T_x_ MXene has been explored to enhance room-temperature NH_3_ gas-sensing performance over a concentration range of 5 to 500 ppm [36]. In one study, Ti_3_C_2_T_x_ MXene was successfully alkalized using 2/5/8M NaOH solutions for 12 h, which significantly improved its rapid response/recovery characteristics. The alkalization process promoted the intercalation of Na^+^ ions between the Ti_3_C_2_T_x_ MXene nanosheets, facilitating the replacement of –F termination groups with more hydrophilic –OH terminations, which produced additional active sites for NH_3_ adsorption. When exposed to 200 ppm NH_3_, the alkalized Ti_3_C_2_T_x_ MXene exhibited faster response/recovery times (73/38 s) compared to pure Ti_3_C_2_T_x_ MXene (83/84 s) at 40% relative humidity and room temperature. The alkalized samples demonstrated excellent cyclic stability over five response/recovery cycles and long-term stability for 17 days, but pristine Ti_3_C_2_T_x_ MXene showed a gradual decline in sensing performance. As a result, the alkalized Ti_3_C_2_T_x_ MXene achieved a high response of 100.3% at 500 ppm with LOD of 5 ppm. The enhanced sensing mechanism was attributed to the interaction of NH_3_ molecules with –OH and –O terminations along with H_2_O-assisted electron donation towards the Ti_3_C_2_T_x_ MXene, contributing to an increase in sensor resistance. Overall, the strategic alkalization approach effectively improved the room-temperature NH_3_-sensing capability of Ti_3_C_2_T_x_ MXene.

Following a similar strategy, Zhang et al. [37] modified the Ti_3_C_2_T_x_ MXene surface through KOH alkalization followed by annealing under nitrogen atmosphere to enhance NH_3_ gas-sensing performance. These combined effects increased the surface –OH and –O termination groups and expanded the interlayer spacing from 9.1 to 12.1 A°, which effectively facilitated the NH_3_ adsorption and diffusion within the Ti_3_C_2_T_x_ MXene nanosheets. After the annealing process, the characteristic layered morphology of Ti_3_C_2_T_x_ MXene was preserved, accompanied by improved crystallinity and grain growth. As a result, the annealed Ti_3_C_2_T_x_ MXene exhibited a superior room-temperature NH_3_ response of 35.7 at 200 ppm, which was higher than that of alkalized (30.1) or pure Ti_3_C_2_T_x_ MXene (18.7) with response times of 170, 300, and 200 s, respectively. Initially, alkalization improved the interlayer spacing and generated abundant reactive sites, which contributed to improved NH_3_ selectivity and adsorption. The heat-treated samples achieved superior NH_3_ gas sensing compared with the alkalized Ti_3_C_2_T_x_ MXene. For annealed samples, linear increment in the O/F ratio and interlayer spacing advanced the NH_3_ gas-sensing performance even after 50 days, with a response of 36.11 at 5 ppm. These findings further demonstrate the significant role of alkalization and post-treatment strategies in advancing the room-temperature NH_3_ gas-sensing performance of Ti_3_C_2_T_x_ MXene.

### 4.4. Partially Oxidized Ti_3_C_2_ MXene

Controlled oxidation of Ti_3_C_2_T_x_ MXene (at 280 °C) significantly enhanced room-temperature NH_3_ gas-sensing performance [38]. Specifically, the improved sensing behavior was attributed to (i) enhancement of hydrogen bonding between NH_3_ molecules and surface termination groups (–OH and –O) and (ii) synergistic interaction between Ti_3_C_2_T_x_ MXene and in situ formed TiO_2_ (p-n junction), which promoted efficient charge transport behavior during NH_3_ sensing. The abundant surface termination groups provided active adsorption sites for NH_3_ molecules, while the preserved layered structure in Ti_3_C_2_T_x_ MXene facilitated rapid electron transport. The XPS O 1s spectra revealed adsorbed oxygen, –O, and –OH termination groups located at 530.3, 532.1, and 534 eV, respectively. Notably, the oxidation process did not disrupt the intrinsic layered morphology of the Ti_3_C_2_T_x_ MXene, thereby retaining its excellent electrical conductivity. As a result, the oxidized Ti_3_C_2_T_x_ MXene exhibited a high response of 21.3% at 10 ppm NH_3_. At a higher concentration of 500 ppm, the sensor achieved further improvement, with a response of 147% and response/recovery times of 67/157 s. The sensing mechanism involved the reaction of NH_3_ molecules with adsorbed O_2_^−^ (in air) on the sensor surface, which released electrons towards the material, reduced the width of the electron depletion layer, and decreased the sensor resistance. The formation of TiO_2_ (i) increased the charge transfer through the p-n heterojunction, (ii) provided abundant oxygen-related defects to increase NH_3_ adsorption, and (iii) created active sites for the stabilization of the Ti_3_C_2_T_x_ MXene for higher NH_3_-sensing capability. The oxidized Ti_3_C_2_T_x_ MXene showed a negligible response (2%) towards ethanol at 500 ppm. Responses of 138, 71, 56, 47, and 41% (at 500 ppm) were observed at various relative humidity conditions of 28, 42, 50, 62, and 70%, respectively. It should be noted that the response level was relatively stable under higher humid conditions (62 to 70%). Overall, the controlled oxidation process effectively optimized the surface chemistry and interfacial charge transportation of the Ti_3_C_2_T_x_ MXene, which enabled highly sensitive room-temperature NH_3_ detection with LOD of 10 ppm.

### 4.5. Ti_3_C_2_ MXene/Sulfur

**Figure 4:**
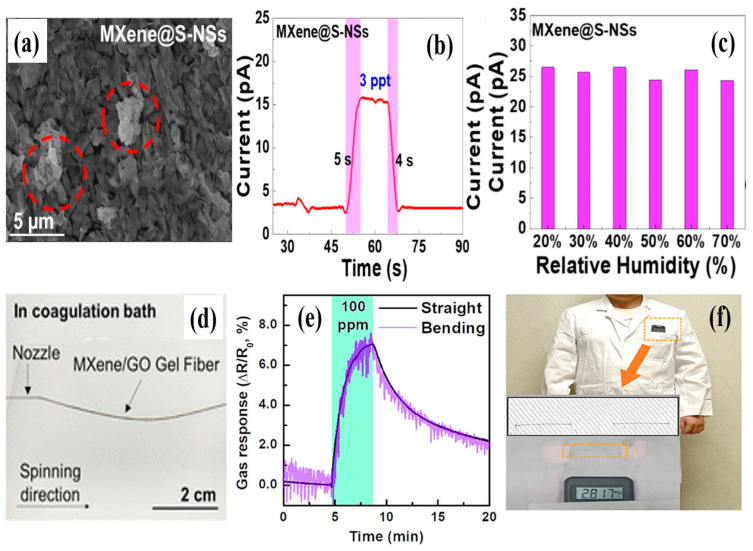
(**a**) Surface morphology of sulfur nanosheets integrated Ti_3_C_2_ MXene, (**b**) ultrafast response and recovery times of about 5 and 4 s, (**c**) stable NH_3_ gas sensing under relative humidity ranging from 20 to 70% (Reprinted from Ref. [39], copyright with permission from Elsevier), (**d**) formation of Ti_3_C_2_T_x_/graphene flexible gel fiber, (**e**) unaltered NH_3_ gas-sensing performance of Ti_3_C_2_T_x_/graphene composite under straight and bent conditions, (**f**) flexible Ti_3_C_2_T_x_ MXene/graphene hybrid fibers woven into a laboratory coat (Reprinted from Ref. [40], copyright with permission from ACS Publication).

Shen et al. [39] demonstrated ultra-sensitive NH_3_ gas sensing at the ppt level (3, 5, 7, 10, and 15) by integrating Ti_3_C_2_ MXene with sulfur nanosheets. The interlayer spacing of Ti_3_C_2_ MXene nanosheets (2–3 μm) was effectively covered by smaller sulfur nanosheets with sizes ranging from 200 to 300 nm. With an increase in NH_3_ concentration from 3 to 15 ppt, the sensing response exhibited linear enhancement accompanied by rapid adsorption and desorption kinetics. The integration of sulfur nanosheets with Ti_3_C_2_ MXene nanosheets is shown in Figure 4a. Notably, the response/recovery times of Ti_3_C_2_ MXene@sulfur nanosheets (5/4 s) were significantly lower than those of pure sulfur nanosheets (60/18 s). The superior electrical conductivity of Ti_3_C_2_ MXene compared with sulfur nanosheets improved the charge transfer and gas-sensing performance at the ppt level. Thus, the Ti_3_C_2_ MXene@sulfur composite exhibited remarkable NH_3_ sensing by achieving a response current of about 15 pA (sulfur nanosheets-3 pA) with ultra-rapid response and recovery times of 5 and 4 s, respectively (Figure 4b). Under these conditions, a sensing response of 375% was achieved at NH_3_ concentration of 3 ppt. This structural integration again proved cyclic stability (across five cycles) and long-term stability over 20 days at 3 ppt level. The composite architecture also maintained a stable NH_3_ response under various relative humidity conditions (20–70%), as shown in Figure 4c. In that study, water molecules did not interfere with NH_3_ adsorption sites under relative humidity conditions between 20 to 70%. At higher conditions (>80%), excessive H_2_O molecules compete with NH_3_, thereby reducing the availability of adsorption sites. In this system, NH_3_ molecules acted as electron donors, which facilitated charge transfer at the Ti_3_C_2_ MXene@sulfur interface and resulted in improved sensing performance. The enhanced NH_3_ sensing mechanism was mainly attributed to the synergistic effects of sulfur vacancies, high electrical conductivity of Ti_3_C_2_ MXene, and surface termination groups, which promoted efficient adsorption and desorption of NH_3_ molecules even under highly humid conditions.

### 4.6. Ti_3_C_2_ MXene/Graphene

The integration of two carbon-based materials (Ti_3_C_2_T_x_ MXene and graphene) into a wearable and flexible fiber architecture demonstrated efficient room-temperature NH_3_ gas sensing [40]. Figure 4d shows a photograph of the Ti_3_C_2_T_x_/rGO flexible gel fiber. The synergistic interaction between Ti_3_C_2_T_x_ MXene and rGO sheets effectively suppressed the severe aggregation of Ti_3_C_2_T_x_ MXene nanosheets at the molecular level under pH 6 conditions, which resulted in a well-defined hybrid structure. At selective content of about 40% Ti_3_C_2_T_x_ MXene, significant enhancement in the number of active adsorption sites for NH_3_ molecules was achieved. In particular, the density of Ti–O active sites in Ti_3_C_2_T_x_ MXene increased three-fold after strategic hybridization with graphene. Accordingly, the resultant composite facilitated stronger NH_3_ adsorption and improving the sensing performance. Thus, the dominance of Ti_3_C_2_T_x_ MXene and Ti–O active sites achieved a high response of 7.21% toward 100 ppm NH_3_, as shown in Figure 4e. The selectivity towards ethanol was very low at about 1%, which suggests preferential adsorption of NH_3_ molecules by the Ti_3_C_2_T_x_ MXene//rGO hybrid fibers. In addition, the sensor achieved LOD of 10 ppm with response of 4.26%. The mechanically robust Ti_3_C_2_T_x_ MXene//rGO hybrid fibers maintained consistent gas-sensing performance under repeated bending and releasing cycles. The practical demonstration of wearable Ti_3_C_2_T_x_/graphene hybrid fiber integrated into a laboratory coat is presented in Figure 4f. Overall, the synergistic combination of Ti_3_C_2_T_x_ MXene and graphene in a flexible fiber architecture provided an effective strategy for the advancement in next-generation wearable NH_3_ gas sensors operating at room temperature.

**Figure 5:**
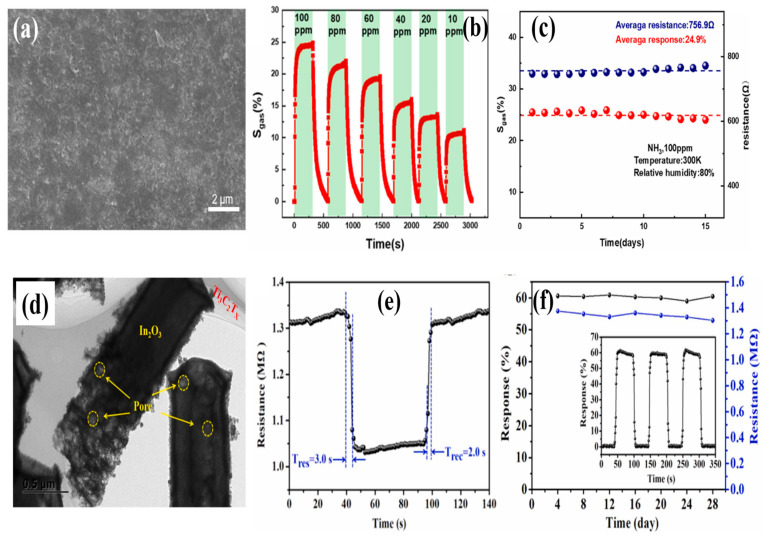
(**a**) Surface morphology of the Ti_3_C_2_T_x_/graphene composite film, (**b**) NH_3_ gas response over a concentration range of 10–100 ppm, (**c**) remarkable long-term stability of the In_2_O_3_/Ti_3_C_2_T_x_ MXene composite with a stable response over 16 days at 80% relative humidity, (Reprinted from Ref. [41], copyright with permission from Elsevier), (**d**) TEM image of In_2_O_3_ microtubes interfaced with Ti_3_C_2_T_x_ MXene, (**e**) ultrafast response and recovery times of 3 s and 2 s for the In_2_O_3_/Ti_3_C_2_T_x_ MXene composite, (**f**) excellent cyclic and long-term stability of the In_2_O_3_/Ti_3_C_2_T_x_ MXene composite, (Reprinted from Ref. [44], copyright with permission from Elsevier).

Li et al. [41] significantly improved the room-temperature NH_3_ gas-sensing performance of Ti_3_C_2_T_x_/graphene composite compared to pure Ti_3_C_2_T_x_ and graphene. The authors attributed the enhanced sensing characteristics to the abundant surface termination groups on the Ti_3_C_2_T_x_/graphene surface, which promoted the adsorption of NH_3_ molecules. In particular, the –O and –OH termination groups effectively interacted with adsorbed NH_3_ molecules, which released three electrons and one electron, respectively. The surface morphology of the Ti_3_C_2_T_x_/graphene composite film is shown in Figure 5a. As a p-type sensing material, the Ti_3_C_2_T_x_/graphene composite accepted electrons from NH_3_ molecules, which led to increase in sensor resistance. The sensing response was investigated over an NH_3_ concentration range of 0.5 to 100 ppm, exhibiting a linear increment from 2.0 to 25.5%, respectively, as shown in Figure 5b. The response towards NO_2_ (5.4%) and H_2_ (3.7%) was lower compared to NH_3_, which confirmed the developed composite’s high selectivity towards NH_3_. At 100 ppm NH_3_, the composite exhibited response and recovery times of 26 and 148 s, respectively. Furthermore, the Ti_3_C_2_T_x_/graphene sensor demonstrated excellent repeatability over three consecutive sensing cycles, with a stable response of 25% and outstanding long-term stability, maintaining an average response of 24.9% after 16 days under 80% relative humidity (Figure 5c). The hydrophilic behavior favored stable NH_3_ sensing at higher humidity conditions. These results successfully demonstrated that the synergistic integration between p-type Ti_3_C_2_T_x_ MXene and graphene substantially enhanced the room-temperature NH_3_-sensing performance under highly humid environmental conditions.

### 4.7. Ti_3_C_2_ MXene/Semiconductors

Furthermore, He et al. [42] enhanced the room-temperature NH_3_ gas-sensing performance by decorating Ti_3_C_2_T_x_ MXene nanosheets with SnO_2_ nanoparticles. The incorporation of SnO_2_ nanoparticles increased the amount of surface-adsorbed oxygen species, which created additional active sites for NH_3_ adsorption and facilitated improvement in gas sensing performance. Interestingly, the Ti_3_C_2_T_x_/SnO_2_ composite exhibited the highest sensing performance at room temperature, whereas the sensor showed almost no response at 100 °C. A linear decrease in sensor response was observed as the operating temperature increased from 25 to 100 °C. This deterioration in sensing performance at elevated temperatures was attributed to the gradual reduction in hydroxyl (–OH) surface terminations. The O 1s peak showed integrated lattice oxygen (530.1 eV) and adsorbed oxygen (532 eV). The improvement in surface adsorbed oxygen created more active sites, increasing sensing performance. Accordingly, the Ti_3_C_2_T_x_/SnO_2_ composite achieved an excellent response of 40% toward 50 ppm at room temperature under relative humidity of 46%, with response and recovery times of 36 and 44 s, respectively. The introduction of SnO_2_ increased NH_3_ exposure towards the interfacial regions, density of reactive oxygen species on the surface, and baseline stability. Furthermore, the response towards ethanol was very low (3.12%), which confirmed high selectivity towards NH_3_. The sensor also exhibited an ultralow LOD of 4.29 ppb, demonstrating its capability for highly sensitive NH_3_ detection. In addition, the composite maintained stable sensing performance for 60 days at NH_3_ concentration of 50 ppm, indicating excellent long-term stability. The electrons transfer from NH_3_ to Ti_3_C_2_T_x_ increased the sensor resistance and NH_3_ sensing capacity. As the relative humidity increased from 25 to 85%, the response was higher at moderate conditions (45% humidity) under increased NH_3_ adsorption and reaction pathways. Overall, the synergistic interaction between Ti_3_C_2_T_x_ and SnO_2_ nanoparticles significantly enhanced NH_3_ adsorption, which enabled superior room-temperature sensing performance, high tolerance of humidity, and excellent long-term operational stability.

Yao et al. [43] synthesized Ti_3_C_2_T_x_ MXene/SnO nanocomposite by integrating ultrathin SnO nanoflakes with Ti_3_C_2_T_x_ MXene through a one-step hydrothermal method, for room-temperature NH_3_ vapor response over a wide range of 1 to 1000 ppm concentrations. The formation of p-n heterojunctions improved the charge carrier concentration, which led to superior NH_3_-sensing performance compared to individual p-type or n-type components. Moreover, the matrix effectively suppressed the oxidation of SnO into SnO_2_, preserving the structural and chemical stability of the composite. Furthermore, the conductive Ti_3_C_2_T_x_ skeleton increased the surface area of the composite (14.27 m^2^/g), which generated abundant oxygen adsorption sites and active reaction centers for NH_3_ molecules. In addition to lattice oxygen (530.3 eV), formation of surface adsorbed oxygen species at 531.8 eV was noticed. Here, the adsorbed oxygen species mainly contributed to the improved gas-sensing performance. In addition, the ultrathin SnO nanoflakes with a thickness of 9 nm facilitated rapid electron transportation and accelerated the sensing kinetics. The synergistic interaction between Ti_3_C_2_T_x_ MXene and SnO effectively modulated the electrical conductivity of the p-n heterojunctions, which resulted enhanced response and recovery characteristics. Furthermore, SnO greatly contributed to increased surface reactions through adsorbed oxygen, charge transfer through heterostructure, and abundant surface-active sites to achieve long-term sensing stability. The nanocomposite exhibited a response of 7.8 for 200 ppm (35.49 for 1000 ppm), with response and recovery times of 61 and 119 s, respectively. The response of about 1.59 towards ethanol was very low compared to NH_3_, which confirmed high selectivity towards NH_3_. Furthermore, the sensor achieved LOD of 1 ppm and demonstrated excellent repeatability over four sensing cycles and stable long-term performance for 22 days. The interaction of NH_3_ with adsorbed oxygen transferred the electrons towards the Ti_3_C_2_T_x_ MXene/SnO, improving the NH_3_ sensing performance. According to the gas-sensing signal direction, SnO behaved like an n-type semiconductor, which created p-n junction at the interface of p-type Ti_3_C_2_T_x_ MXene. Overall, the Ti_3_C_2_T_x_ MXene/SnO heterostructure exhibited a broad NH_3_-detection range (1–1000 ppm), high sensitivity, rapid sensing kinetics, and excellent long-term stability for advancing the room-temperature NH_3_ sensing applications.

Achieving ultrafast response and recovery is crucial for realizing high-performance gas sensors with rapid adsorption and desorption kinetics. In this context, Liu et al. [44] developed a metal–organic frameworks (MOFs)-derived In_2_O_3_ microtube (2–5 μm)/Ti_3_C_2_T_x_ MXene heterostructure for room-temperature NH_3_ gas sensing. The TEM image of the MOFs-derived In_2_O_3_ microtubes/Ti_3_C_2_T_x_ MXene heterostructure is shown in Figure 5d. The composite exhibited a highly mesoporous architecture with a large specific surface area of 446.63 m^2^/g and pore size distribution of 5–10 nm, which provided abundant active sites for NH_3_ adsorption. The O 1s spectrum revealed lattice oxygen, oxygen defects, and chemisorbed oxygen at 528.9, 530.5, and 532.0 eV, respectively. These features created more gas-sensing capability. As shown in Figure 5e, the heterostructure achieved ultrafast response and recovery times of 3 and 2 s toward 5 ppm NH_3_ at room temperature, whereas the corresponding values increased to 28 and 65 s at 200 °C. The significantly shortened response time at room temperature indicated the enhancement in NH_3_ adsorption kinetics and efficient electron transfer within the heterostructure. However, at 100 °C, the response/recovery time slightly increased to 5/3 s due to the partial oxidation of the Ti_3_C_2_T_x_ MXene, which reduced the electrical conductivity of the composite. The NH_3_ molecules interacted with adsorbed oxygen and released electrons to the In_2_O_3_/Ti_3_C_2_T_x_ heterostructure, reducing the sensor resistance. Introduced In_2_O_3_ behaved like an active sensing component for the detection of NH_3_ through surface adsorption. Due to its mesoporous architecture, In_2_O_3_ provided numerous active sites at the interface of Ti_3_C_2_T_x_ MXene, leading to enhancement in charge transfer and sensor response at room temperature. Thus, the MOFs-derived In_2_O_3_/Ti_3_C_2_T_x_ MXene composite exhibited outstanding room-temperature NH_3_ gas sensing of 60.6% with ultra-fast response and recovery characteristics (Figure 5f). The response towards NH_3_ was significantly higher than ethanol, CO, and NO_2_, which suggests high selectivity towards NH_3_. Furthermore, the sensor retained a stable NH_3_ response up to relative humidity of 60%, demonstrating excellent humidity tolerance. At higher relative humidity conditions (>60%), decreases in response were noticed due to the interference of water molecules. The work function of p-type Ti_3_C_2_T_x_ MXene (4.28 eV) was higher than n-type In_2_O_3_ (4.28 eV). Thus, electrons transferred from In_2_O_3_ to the surface functional groups modulated the p-type Ti_3_C_2_T_x_ MXene. Overall, the formation of p-n heterojunctions between In_2_O_3_ and Ti_3_C_2_T_x_ MXene significantly enhanced charge transfer and gas-adsorption features for exceptional room-temperature NH_3_-sensing performance.

The interaction of NiO with a Ni_2_P nanosheet heterostructure enhanced H_2_S gas sensing [45]. In this context, Yang et al. [46] highlighted the incorporation of NiO nanoparticles into Ti_3_C_2_T_x_ MXene (NiO/Ti_3_C_2_T_x_) for room-temperature NH_3_ gas sensing. The NiO/Ti_3_C_2_T_x_ heterostructure exhibited faster recovery than response over an NH_3_ concentration range of 10–50 ppm. More importantly, the sensing response of NiO/Ti_3_C_2_T_x_ composite was 1.18 and 1.38 times higher than those of pristine Ti_3_C_2_T_x_ MXene and NiO, respectively. This enhanced sensing performance was attributed to the increased density of active adsorption sites and improved electron transport across the NiO/Ti_3_C_2_T_x_ heterojunction. Furthermore, pristine Ti_3_C_2_T_x_ MXene exhibited a higher response than pure NiO owing to its abundant surface termination groups (–O, –OH, and –F), which provided favorable adsorption sites for NH_3_ molecules. According to the O 1s spectra, Ni-O and –OH groups on the NiO corresponded to lattice oxygen and adsorbed oxygen, respectively. The effectively adsorbed NH_3_ molecules interacted with adsorbed oxygen and transferred electrons towards the sensor, increasing the sensor resistance. Specifically, NiO increased the NH_3_ adsorbed oxygen species, surface area, and Schottky heterojunction, increasing the adsorption sites for room-temperature NH_3_ gas sensing. Upon forming the heterostructure, the NiO/Ti_3_C_2_T_x_ composite achieved a response of 6.13% toward 50 ppm NH_3_ with rapid response and recovery times of 60 and 19 s, respectively. Responses towards ethanol and IPA were observed to be 2.26 and 0.53%, respectively, which suggests high selectivity towards NH_3_ (6.13%). The sensor was also capable of detecting NH_3_ concentrations as low as 10 ppm and demonstrated excellent repeatability over 15 sensing cycles at 50 ppm (long-term stability for 15 days). In addition, the NiO nanoparticles increased the specific surface area and number of active sites available for oxygen and NH_3_ adsorption. The NH_3_ response was highest at 10% humidity, gradually decreasing at 30 to 50%, with a significant reduction between 70 to 90% relative humidity conditions. Combined with the abundant surface termination groups (–O, –OH, and –F) and high charge-carrier mobility in Ti_3_C_2_T_x_ MXene, enhanced room-temperature NH_3_-sensing performance was achieved in the NiO/Ti_3_C_2_T_x_ heterostructure.

In addition to previously reported SnO_2_ [42], SnO [43], and NiO [46] semiconductor nanostructures, Pasupuleti et al. [47] demonstrated sub-ppb-level NH_3_ detection at room temperature under UV illumination using a ZnO@Ti_3_C_2_T_x_ MXene composite. In this composite, ZnO nanoparticles were uniformly anchored onto Ti_3_C_2_T_x_ MXene nanosheets, which resulted in a larger specific surface area (47.55 m^2^/g) than that of pristine ZnO (23.61 m^2^/g) and Ti_3_C_2_T_x_ (13.86 m^2^/g). The increased surface area provided abundant active sites for NH_3_ adsorption, which facilitated both sensitivity and selectivity. Key features such as lattice oxygen, oxygen vacancy, and chemisorbed oxygen were observed in the O 1s spectrum, and these promoted NH_3_ adsorption. Furthermore, the Schottky junction formed at the ZnO/Ti_3_C_2_T_x_ interface reduced the electron depletion layer upon NH_3_ exposure, which played a crucial role in enhancing the sensing performance. Introduction of ZnO nanoparticles created oxygen vacancies (active adsorption sites), Schottky junctions (charge transfer), and suppressed Ti_3_C_2_T_x_ nanosheet restacking (high surface area), increasing the long-term stability of NH_3_ gas sensing. Under UV illumination, photogenerated holes promoted the neutralization of adsorbed O_2_^−^ ions, promoting the desorption of NH_3_ molecules and accelerating the recovery process. As a result, the ZnO@Ti_3_C_2_T_x_ exhibited response and recovery times of 92 and 104 s, respectively, with an impressive LOD of 89.41 ppb. At an NH_3_ concentration of 20 ppm, the sensor achieved a remarkable response of 39.16%, higher than for CO (6.415) and NO_2_ (16.25%) gases. These findings demonstrated that ZnO nanoparticles effectively modulated the interfacial electronic structure of Ti_3_C_2_T_x_ MXene for efficient photo-assisted room-temperature NH_3_ gas sensing.

**Figure 6:**
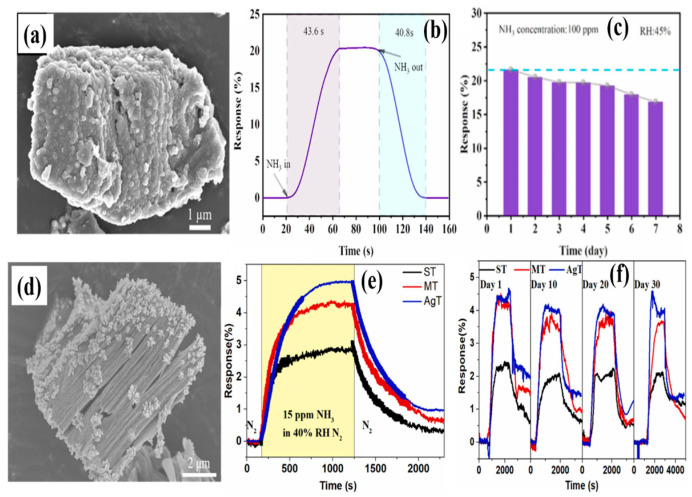
(**a**) Surface morphology of the Ti_3_C_2_T_x_ MXene/MoS_2_/PPy composite, (**b**) response and recovery times of 43.6 and 40.8 s with a response of 21% under 100 ppm NH_3_, (**c**) long-term stability over 7 days at 45% relative humidity (Reprinted from Ref. [48], copyright with permission from Elsevier), (**d**) surface morphology of Ag nanoparticle-integrated oxygen-rich p-type Ti_3_C_2_T_x_ MXene, (**e**) enhanced response toward 15 ppm NH_3_ under 40% relative humidity, and (**f**) remarkable long-term cyclic stability and NH_3_ sensing performance over 30 days (Reprinted from Ref. [51], copyright with permission from Elsevier).

In another study, Lu et al. [48] successively fabricated a Ti_3_C_2_T_x_ MXene/MoS_2_/PPy ternary composite for efficient room-temperature NH_3_ gas sensing. In this hybrid structure, PPy provided abundant hydrogen-bonding active sites for enhanced NH_3_ adsorption, while MoS_2_ nanosheets increased the interlayer spacing of Ti_3_C_2_T_x_ MXene nanosheets for effective exposure of additional active sites and gas adsorption. The surface morphology of the Ti_3_C_2_T_x_/MoS_2_/PPy is shown in Figure 6a. Owing to the p-type semiconducting behavior of Ti_3_C_2_T_x_, electron donation from NH_3_ molecules resulted in increased sensor resistance. The composite exhibited a detectable response of 2.31% at 10 ppm NH_3_ and a high response of 21% at 100 ppm with rapid response/recovery times of 43.6/40.8 s at room temperature under 45% relative humidity (Figure 6b). The response decreased from 23 to 4.7% (at 100 ppm) by increasing the relative humidity from 30 to 90%, respectively. Adsorption of water molecules on active sensing sites prevented the NH_3_ molecules from reaching the sensor surface. The response towards ethanol and NO_2_ was 5.4 and 2.5%, respectively. This suggests that the Ti_3_C_2_T_x_ MXene/MoS_2_/PPy composite was highly selective towards NH_3_ compared with ethanol and NO_2_. In addition, the sensor displayed excellent repeatability over five sensing cycles and maintained stable long-term performance for 7 days under the same humidity conditions (Figure 6c). Overall, the synergistic effects of Ti_3_C_2_T_x_ MXene, MoS_2_ nanosheets, and PPy significantly improved NH_3_ sensing performance in harsh humid environments.

In another study, Bi_2_O_3_ was integrated with Ti_3_C_2_T_x_ (Bi_2_O_3_/Ti_3_C_2_T_x_) for room-temperature NH_3_ gas sensing [49]. This composite was highly selective to NH_3_ with a response of 61% at 100 ppm, compared with ethanol (10%), methanol (3%), and acetic acid (4%). High surface area, electrical conductivity, interfacial charge transfer, and surface functional groups (–O, –OH, and –F) accelerated the NH_3_ adsorption and interaction. Response and recovery times of 61 and 164 s were observed for the Bi_2_O_3_/Ti_3_C_2_T_x_ composite at 100 ppm. Notably, the response linearly increased from 61 to 124% with an increase in relative humidity from 50 to 90%, which suggested high humidity tolerance. Identical response signals were achieved after six cycles and 20 days (five cycles each day), showing long-term stability. Thus, the developed Bi_2_O_3_/Ti_3_C_2_T_x_ composite provided remarkable adsorption/desorption kinetics at the interface with minimal degradation of active sensing sites.

Seekaew et al. [50] developed a Ti_3_C_2_T_x_ MXene/GO/CuO/ZnO (9:1:5:5 wt%) heterojunction composite by hydrothermal process, for room-temperature NH_3_ gas sensing in the range of 25 to 200 ppm. This combination included p-n junctions. The intimate contact between various semiconductors achieved abundant adsorption sites, charge transfer, and oxygen vacancies, thereby enhancing NH_3_ gas sensing. Notably, stable response and complete recovery were achieved with response time and recovery of 26 and 26 s, respectively, at 100 ppm. The LOD was about 4.1 ppm. The presence of –OH and –O groups accelerated the NH_3_ adsorption. Thus, an NH_3_ response of 96.3% was achieved at 200 ppm (25 ppm-30.7%). The response towards NH_3_ was significantly higher than ethanol, methanol, and acetone. No response towards NO_2_ was achieved by the selective composite. These results suggest that the surface chemistry favored NH_3_ selectivity compared with other volatile organic compounds. At 80 to 90% relative humidity, the sensor showed a highly stable response with slight decrease in resistance. At 200 ppm, this composite achieved stable response and recovery curves after six repeated cycles and long-term stability after 45 days. Overall, the formation of multiple-semiconductor-heterojunction-integrated Ti_3_C_2_T_x_ MXene contributed to excellent response and recovery in the detection of NH_3_ at room temperature.

### 4.8. Ti_3_C_2_ MXene/Ag and Au Metals

The incorporation of Ag nanoparticles into oxygen-rich p-type Ti_3_C_2_T_x_ MXene (AgT) significantly enhanced room-temperature NH_3_ gas-sensing performance [51]. The surface morphology of an AgT composite is presented in Figure 6d. During the sensing process, NH_3_ gas molecules were initially adsorbed onto the Ag nanoparticles, followed by their diffusion into the Ti_3_C_2_T_x_ MXene layers, resulting in an increment in the sensor resistance. Interestingly, the rapid recovery behavior was attributed to the abundant surface termination groups, where the –O terminations exhibited weaker adsorption energy and –OH groups facilitated faster NH_3_ desorption. XPS studies showed that lattice oxygen and adsorbed oxygen were observed through Ti–O–Ti formation at 530.0 eV. Furthermore, –O and –OH termination groups were observed through C–Ti–OH formation at 531.9 eV. Accordingly, the AgT composite exhibited enhanced NH_3_ gas-sensing performance up to 10 ppm (stable at 15 and 20 ppm). In this process, the AgT sensor achieved responses of 4.35% and 4.96% toward 10 and 15 ppm NH_3_, respectively, which outperformed pristine single-layer Ti_3_C_2_T_x_ MXene (2.93%) and multilayer Ti_3_C_2_T_x_ MXene (4.30%), as shown in Figure 6e. In addition, the AgT composite achieved LOD of 0.5 ppm. At a relative humidity of 40%, the AgT sensor exhibited response and recovery times of 511 and 605 s, respectively, compared to 319/578 s for single-layer and 446/659 s for multilayer Ti_3_C_2_T_x_ MXene. Moreover, the synergistic effects of the multilayer architecture, Ag nanoparticles, and abundant surface termination groups enabled excellent long-term stability over 30 days, even under 40% relative humidity (Figure 6f). DFT studies highlighted the superior NH_3_ adsorption of Ag-Ti_3_C_2_O_2_ (–O termination groups), with adsorption energy of −0.445 eV (dry) and −0.490 eV (pre-adsorbed H_2_O) at adsorption distances of 2.494 and 2.269 Å, respectively. Pre-adsorbed H_2_O increased the charge transfer to 0.08 e, which confirmed enhanced NH_3_ adsorption and sensing performance compared with pristine Ti_3_C_2_O_2_. Overall, defect engineering combined with surface functionalization through Ag nanoparticles effectively improved the room-temperature NH_3_-sensing performance of Ti_3_C_2_T_x_ MXene.

**Figure 7:**
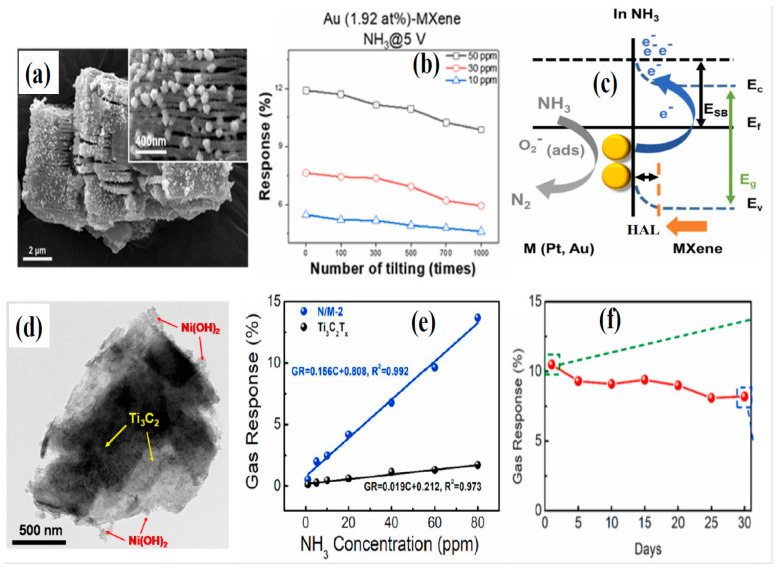
(**a**) Surface morphology of Au nanoparticle-decorated Ti_3_C_2_T_x_ MXene nanosheets, (**b**) NH_3_ gas response over the concentration range of 10–50 ppm at an Au concentration of 1.92%, (**c**) charge transfer mechanism and NH_3_ gas sensing mechanism of the Au/Ti_3_C_2_T_x_ MXene composite, (Reprinted from Ref. [52], copyright with permission from Elsevier), (**d**) TEM image of Ni(OH)_2_ nanoparticles interfaced with Ti_3_C_2_T_x_ MXene nanosheets, (**e**) enhanced gas-sensing response of the Ni(OH)_2_ nanoparticles interfaced with Ti_3_C_2_T_x_ compared with pure Ti_3_C_2_T_x_ MXene, and (**f**) stable long-term response over 30 days at 50 ppm NH_3_ for the Ni(OH)_2_ nanoparticle-interfaced Ti_3_C_2_T_x_ MXene composite (Reprinted from Ref. [56], copyright with permission from Elsevier).

Interestingly, the selective interaction of noble Au (1.92 at%) and Pt (0.83%) metallic nanoparticles with Ti_3_C_2_T_x_ MXene nanosheets for NH_3_ gas-sensing applications was investigated [52]. The sensor thickness was maintained within the range of 10 to 20 μm. Among the two-metal nanoparticle-decorated composites, Au-modified Ti_3_C_2_T_x_ MXene exhibited superior NH_3_ response and selectivity compared to Pt-modified Ti_3_C_2_T_x_ MXene. The surface morphology of the Au nanoparticle-interfaced Ti_3_C_2_T_x_ MXene nanosheets is presented in Figure 7a. Ohmic contact between sensors and electrodes with negligible polarization did not influence the sensing signal. Accordingly, Au (1.92 at%) and Pt (0.83%)-decorated Ti_3_C_2_T_x_ MXene sensors demonstrated room-temperature NH_3_ responses (100 ppm) of 16 and 9%, respectively. The response/recovery times for Au- and Pt-decorated sensors were recorded as 87/217 s and 77/248 s, respectively, over six consecutive sensing cycles. Furthermore, the flexibility of the sensors was evaluated under 0 to 120° bending conditions. Remarkably, after bending at 120°, the flexible NH_3_ gas sensors retained responses of 9.9% and 6% for Au (1.92 at%) and Pt (0.83 at%) nanoparticle-decorated Ti_3_C_2_T_x_ MXene, respectively (Figure 7b). These composite sensors also demonstrated stable NH_3_-sensing performance under highly humid conditions. As an electron-donating gas, NH_3_ molecules effectively transferred electrons towards the sensor, which altered the sensor resistance. The proposed charge transfer mechanism and interaction between NH_3_ molecules and the sensor surface is illustrated in Figure 7c. Overall, the incorporation of noble metal nanoparticles into Ti_3_C_2_T_x_ MXene nanosheets enhanced the sensing characteristics and enabled flexible, room-temperature NH_3_ gas detection.

### 4.9. Ti_3_C_2_ MXene/Polymers

Qiu et al. [53] enhanced room-temperature NH_3_ gas sensing by developing an organic–inorganic p-n heterojunction based on PEDOT:PSS/nitrogen-doped Ti_3_C_2_T_x_ MXene composite film. A selective mass ratio of 1:0.5 (N-MXene:PEDOT:PSS) maintained stable NH_3_ gas-sensing performance owing to the dominant electron extraction by the PEDOT matrix compared to N-MXene. The incorporated N-atoms acted as electron donors, which increased the density of active adsorption sites within the PEDOT:PSS matrix. Furthermore, the formation of TiO_2_ nanoparticles between the N-MXene layers enlarged the interlayer space and suppressed the retacking of MXene nanosheets, which facilitated efficient NH_3_ diffusion and improved response and recovery characteristics. Consequently, the well-defined layered architecture promoted rapid gas transportation, resulting in response and recovery times of 280 and 393.6 s, respectively. Under these optimized conditions, the PEDOT:PSS/N-MXene composite exhibited a response of about 13% toward 10 ppm at 20 °C and 36%RH. The NO_2_ and CO response was negligible compared to NH_3_, which suggests PEDOT:PSS/N-MXene’s high selectivity towards NH_3_ gas. In addition, the sensor demonstrated excellent repeatability over four sensing cycles and maintained stable performance up to 17 days. These results confirmed that the synergistic integration between PEDOT:PSS and N-doped Ti_3_C_2_T_x_ MXene significantly improved the room-temperature NH_3_-sensing performance.

Arkoti et al. [54] reported improvement in NH_3_ gas-sensing performance by developing Ti_3_C_2_T_x_ MXene-based sensors with and without a mixed-matrix membrane (MMM) filter composed of PVDF-ZIF-67. The authors demonstrated that the incorporation of the MMM filter improved the long-term stability of the sensor. In comparison, pure Ti_3_C_2_T_x_ MXene dominated the sensor behavior than with the MMM filter. Interestingly, strong hydrogen-bonding interactions between NH_3_ gas molecules and –OH termination groups influenced the gas-sensing behavior. The minimum detectable NH_3_ concentrations were 9 ppt and 1 ppb for sensors without and with the MMM filter. At 5 ppm NH_3_ concentration, the Ti_3_C_2_T_x_ MXene sensor exhibited response/recovery times of 37.8/34.5 s and 49.5/51.2 s without and with MMM filter, respectively. Furthermore, at 35% relative humidity, the NH_3_-sensing responses of Ti_3_C_2_T_x_ MXene with and without the MMM filter were about 10.6% and 11.86%, respectively. Responses towards ethanol and NO_2_ were observed to be 2.8 and 1.2% without the MMM filter, confirming high selectivity towards NH_3_. The MMM filter facilitated controlled diffusion of NH_3_ molecules toward the Ti_3_C_2_T_x_ MXene layer, resulting in improved long-term stability over 35 days. Overall, the introduction of the MMM filter effectively reduced interference from water molecules and suppressed the oxidation of Ti_3_C_2_T_x_ MXene for enhancing the durability of NH_3_ gas-sensing devices.

Room-temperature NH_3_ gas sensing using PANI/Ti_3_C_2_T_x_ was studied [55]. The formation of –OH and –O functional groups led to effective interaction with NH_3_. The constructive interface between conductive Ti_3_C_2_T_x_ MXene and semiconducting PANI created a potential Schottky barrier, which enhanced the NH_3_ adsorption properties under altered sensor resistance. According to DFT studies, the adsorption energy of NH_3_ (−5.351 eV) was lower than CO (0.057), NO_2_ (−4.498 eV), and ethanol (0.127 eV), which confirmed the remarkable adsorption capacity of NH_3_ on the PANI/Ti_3_C_2_T_x_ surface. Under these conditions, the flexible PANI/Ti_3_C_2_T_x_ composite achieved a room-temperature NH_3_ response of 55.90% at 20 ppm (pure PANI-24.20%). Interestingly, the response increased from 52.32 to 58.54% under relative humidity conditions of 25 and 68%, respectively. This organized flexible PANI/Ti_3_C_2_T_x_ composite achieved excellent cyclic repeatability without decaying even after five cycles. Moreover, long-term stability over 21 days was maintained without altering the response. Overall, remarkable NH_3_ adsorption capacity in the flexible PANI/Ti_3_C_2_T_x_ composite was achieved.

### 4.10. Ti_3_C_2_ MXene/Metal Hydroxides

The incorporation of Ni(OH)_2_ nanoparticles onto Ti_3_C_2_T_x_ MXene nanosheets significantly enhanced room-temperature NH_3_ gas-sensing performance [56]. The introduction of optimized Ni(OH)_2_ loading (7.8 wt.%) increased the interlayer spacing of Ti_3_C_2_T_x_ MXene nanosheets, which provided additional diffusion pathways and abundant adsorption sites for NH_3_ molecules. Consequently, the composite exhibited a high specific surface area of 53.0591 m^2^ g^−1^, which further promoted gas adsorption. The TEM image of the Ni(OH)_2_/Ti_3_C_2_T_x_ MXene composite is presented in Figure 7d. During the hybridization process, some of the –OH surface termination groups were replaced through the incorporation of Ni ions (XPS studies), which modified the surface chemistry of the MXene. Owing to the dominant Ti_3_C_2_T_x_ MXene content relative to low Ni(OH)_2_ loading (7.8 wt.%), electrical resistance of the composite remained primarily governed by the conductive MXene network and enhanced the surface reactivity introduced by Ni(OH)_2_. Thus, the Ni(OH)_2_/Ti_3_C_2_T_x_ composite exhibited a room-temperature NH_3_ response of 13.69% at 80 ppm, as shown in Figure 7e. Furthermore, the sensor demonstrated stable response and recovery times of 78 and 108 s, with a response of 10.3% after five consecutive sensing cycles and excellent long-term stability over 30 days at 50 ppm, as shown in Figure 7f. The response value towards 70 ppm NO (2.4%) was lower than towards NH_3_ (11.6%), which suggests that the Ni(OH)_2_/Ti_3_C_2_T_x_ composite was highly selective towards NH_3_. Notably, the sensor maintained excellent sensing performance under humid conditions, exhibiting responses of 5.1 and 6.2% toward 10 ppm NH_3_ at relative humidities of 40 and 60%, respectively. At higher humidity of 80%, the response decreased to 3.9% due to the excessive water molecules blocking the NH_3_ adsorption sites. The adsorbed NH_3_ transferred electrons to the Ni(OH)_2_ and Ti_3_C_2_T_x_, leading to a decrease in the concentration of holes, which increased the sensor resistance and increased the NH_3_ sensing capacity. Overall, the synergistic integration of Ni(OH)_2_ nanoparticles with Ti_3_C_2_T_x_ MXene effectively enhanced the NH_3_ adsorption, gas diffusion, and humidity tolerance.

Similarly, Huang et al. [57] developed a Co(OH)_2_/Ti_3_C_2_T_x_ nanocomposite for room-temperature NH_3_ gas sensing. The incorporation of optimized Co(OH)_2_ content (2.13%) along with NaOH interaction (alkalization) altered the characteristic (002) diffraction peak position of Ti_3_C_2_T_x_ MXene towards lower diffraction angles, which indicated an increase in the interlayer spacing [35]. This structural modification promoted the diffusion of NH_3_ molecules into the enlarged interlayer channels. In addition, the specific surface area of the Co(OH)_2_/Ti_3_C_2_T_x_ nanocomposite (19.0984 m^2^/g) was substantially higher than that of pure MXene (3.9900 m^2^/g), providing a greater number of active adsorption sites for NH_3_ molecules. Lattice oxygen in the Co(OH)_2_/Ti_3_C_2_T_x_ nanocomposite was observed, with O–H and Ti–O bonds at 529.8 and 531.1 eV, respectively. As a result, the nanocomposite containing 2.13% Co(OH)_2_ exhibited NH_3_ gas response of 14.7% at 100 ppm, with response and recovery times of 39 and 49 s, respectively. The response towards ethanol was negligible a 0.7%, which confirmed the potential response and selectivity of Co(OH)_2_/Ti_3_C_2_T_x_ composite towards NH_3_. Furthermore, the sensor demonstrated excellent repeatability over five sensing cycles and maintained stable sensing performance up to 30 days. The synergistic interaction between Co(OH)_2_ and Ti_3_C_2_T_x_ also enhanced the NH_3_ sensing performance under humid conditions of 30% (high response), 40–60% (decreasing), and 70% (increase again). At higher humidity conditions of 70%, NH_3_ interacted with water molecules and formed hydrated NH_3_ molecules, which increased the Ti_3_C_2_T_x_ nanosheets’ interlayer spacing and sensing ability. The adsorbed NH_3_ transferred the electrons to the Co(OH)_2_ and Ti_3_C_2_T_x_, which reduced the concentration of holes. Overall, the surface modification of Ti_3_C_2_T_x_ MXene with Co(OH)_2_ effectively enhanced the selectivity and room-temperature NH_3_ sensing performance. 

### 4.11. Ti_3_C_2_ MXene/MOFs

Very recently, Qiu et al. [58] successfully demonstrated ppb level NH_3_ gas sensing at room temperature by integrating Ni_3_(HITP)_2_ MOF and Ti_3_C_2_ MXene. In this hybrid structure, Ni_3_(HITP)_2_ MOF crystals were directly grown on the Ti_3_C_2_ MXene surface, enabling intimate interfacial contact between two components. The resulting Ni_3_(HITP)_2_/Ti_3_C_2_ composite exhibited a high specific surface area of 431.2 m^2^/g, which provided abundant active sites for NH_3_ adsorption, diffusion, and transportation. This unique architecture significantly advanced the molecular capture capability of the sensing platform. Furthermore, the formation of Ti–O–Ni and/or Ti–F–Ni bonds at the Ni_3_(HITP)_2_ MOF/Ti_3_C_2_ MXene interface facilitated interfacial charge transfer, improving the gas-sensing performance. This structural integration enhanced the NH_3_ gas-sensing response to 33% at 30 ppm, with rapid response/recovery times of 9/13 s, which demonstrated efficient and reversible NH_3_ adsorption and desorption processes. Also, composite sensors demonstrated excellent cyclic stability over five cycles under NH_3_ concentrations of 1, 5, and 15 ppm, along with long-term stability for more than 50 days at 15 ppm at 30% relative humidity. A remarkably LOD of 100 ppb at room temperature was achieved. After NH_3_ exposure, NH_3_ gas molecules behaved like electron donors and experienced potential redox reaction with adsorbed oxygen ions. Overall, the integration of MOF and Ti_3_C_2_ MXene provided a promising strategy for developing highly sensitive, stable, and room-temperature NH_3_ gas sensors with ppb-level detection capability.

### 4.12. Ti_3_C_2_ MXene/Single Atoms

In addition to the above studies, Ni single-atom active sites coordinated with N and C (Ni–N–C) on Ti_3_C_2_T_x_ MXene (Ni–N–C/Ti_3_C_2_T_x_) have been studied with regard to room-temperature NH_3_ gas sensing [59]. A composite integrated with a gold electrode (AuE-Ni–N–C/Ti_3_C_2_T_x_) exhibited a response of 20.1% toward 5 ppm, with LOD of 29.3 ppb. Furthermore, Ti_3_C_2_T_x_-MXene (ME) and Ti_3_C_2_T_x_-MXene/NMP/PEDOT:PSS (MNPE) electrode inks were employed to fabricate ME-Ni–N–C/Ti_3_C_2_T_x_ and MNPE-Ni–N–C/Ti_3_C_2_T_x_ sensors, which achieved responses of 33.2 and 27.3% at 5 ppm, respectively. The ME-Ni–N–C/Ti_3_C_2_T_x_ and MNPE-Ni–N–C/Ti_3_C_2_T_x_ composites exhibited response/recovery times of 30/80 s and 30/150 s with LOD of 27.0 and 12.1 ppb, respectively. This improved NH_3_ sensing performance was attributed to the oxygen-enriched groups in the MNPE and ME electrodes, which facilitated higher density of surface-active sites than the AuE-Ni–N–C/Ti_3_C_2_T_x_. Moreover, the ME-Ni–N–C/Ti_3_C_2_T_x_ sensor demonstrated superior long-term stability by retaining 35.8% of its sensing response after 4 weeks. These findings highlight the effectiveness of ME and MNPE electrode inks for developing flexible and high-performance NH_3_ gas sensors.

### 4.13. Ti_3_C_2_ MXene/Cationic Polyacrylamide

Rapid NH_3_ gas response/recovery process was achieved by developing flexible cationic polyacrylamide (CPAM)=interfaced Ti_3_C_2_T_x_ MXene nanosheets (CPAM/Ti_3_C_2_T_x_) [60]. The structural defects and surface termination groups (–OH, –O, and –NH_2_) in the CPAM/Ti_3_C_2_T_x_ composite played a crucial role in enhancing the adsorption of NH_3_ gas molecules. Among these functional groups, the bond length (1.89 A°) between the NH_3_ and –OH terminations at adsorption energy of −0.515 eV contributed to Ti_3_C_2_T_x_ MXene’s role in the NH_3_ sensing mechanism. The strong hydrogen bonding between the –OH termination groups and NH_3_ molecules facilitated electron transfer from NH_3_ to CPAM/Ti_3_C_2_T_x_. In this context, the sensor exhibited rapid response/recovery time of 12.7/14.6 s and response of 3.1% at 150 ppm, which further increased to 4.7% at 200 ppm. The presence of abundant functional groups also enabled stable NH_3_-sensing performance at 200 ppm at a relative humidity of 35%. The sensor achieved high tolerance at relative humidity of under 45%, with negligible changes, and 50% decreased response at relative humidity of 95%. Furthermore, CPAM acted as an effective interfacial binder, which promoted strong adhesion between the Ti_3_C_2_T_x_ MXene nanosheets and mechanical stability, ensuing a stable response at 60 °C over 140 operating cycles. Overall, the synergistic effect of surface termination groups and the CPAM interface significantly enhanced the flexibility, stability, and rapid room-temperature NH_3_-sensing performance of the CPAM/Ti_3_C_2_T_x_ composite.

Overall, Ti_3_C_2_T_x_ MXene-based composites exhibited remarkable room-temperature NH_3_ gas sensing, with abundant surface terminations (–OH, –O, and –F), enlarged interlayer spacing, gas diffusion, and adsorption. Functionalization with polymers, graphene, metal oxides, MOFs, noble metals, sulfur nanosheets, and hydroxides greatly enhanced the key features such as adsorption, charge transfer, selectivity, and stability by suppressing oxidation. Results revealed NH_3_ responses up to 147%, ultrafast response/recovery as low as 3/2 s, LOD from ppm to ppt levels, stable operation under humid conditions, bending, and long-term stability. Interestingly, strategies such as oxidation, alkalization, thickness control, and defect engineering further optimized the active sites, enabling highly sensitive, flexible, and wearable NH_3_ gas-sensing platforms at room temperature. Developments in Ti_3_C_2_T_x_ MXene-based composites for room-temperature NH_3_ gas sensing are presented in Table 2.

## 5. Stability of Ti_3_C_2_ MXene-Based Composites Under Humid Conditions

**Figure 8:**
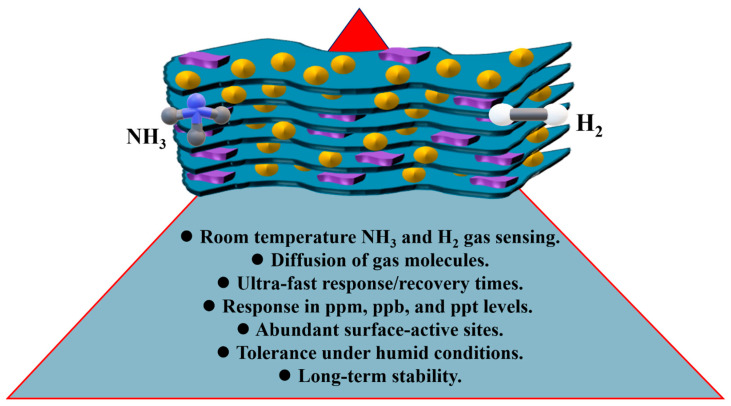
Key features contributing to the enhanced room-temperature NH_3_ and H_2_ gas-sensing performance of Ti_3_C_2_MXene-based nanostructures.

Achieving a stable composite with rapid adsorption/desorption kinetics for target molecules is highly desirable for practical detection of toxic gas at room temperature. The unique advantages of Ti_3_C_2_ MXene, including its layer structure, tunable surface termination groups, and excellent electrical conductivity have significantly progressed the use of Ti_3_C_2_ MXene-based composites for NH_3_ gas sensing across a range of ppm to ppt levels. More importantly, these composites have demonstrated reliable room-temperature NH_3_ sensing performance under harsh environmental conditions, particularly in humid atmospheres. The coordination of Ni(OH)_2_/Ti_3_C_2_T_x_ composite exhibited enhanced NH_3_ responses of 5.1 and 6.2% at 10 ppm under humidity levels of 40 and 60%, respectively [56]. Similarly, a Ti_3_C_2_T_x_/SnO_2_ composite achieved room-temperature NH_3_ response of 40% towards 50 ppm NH_3_ at 46% relative humidity with response and recovery times of 36 and 44 s, respectively [42]. Furthermore, a p-n heterojunction-based MOFs-derived In_2_O_3_/T_i3_C_2_T_x_ MXene composite demonstrated an unaltered NH_3_ response of 60.6% with ultra-fast response/recovery times of 5/3 s [44]. Additionally, Ag nanoparticle-decorated oxygen-rich Ti_3_C_2_T_x_ MXene maintained long-term NH_3_ sensing stability over 30 days even under 40% relative humidity conditions [51]. A p-type Ti_3_C_2_T_x_/graphene composite achieved NH_3_ response of 24.9% under an elevated humidity level of 80% [41]. Interestingly, alkalization treatment of Ti_3_C_2_T_x_ MXene improved the response/recovery characteristics, achieving 73/38 s at 200 ppm NH_3_ under 40% relative humidity [36]. Compared with pristine Ti_3_C_2_T_x_ MXene, the alkalized samples exhibited excellent cyclic stability over five sensing cycles and long-term stability for 17 days without significant response degradation. Furthermore, a Ti_3_C_2_ MXene@sulfur composite demonstrated stable NH_3_ sensing performance over a broad humidity range of 20–70% with ultrafast response and recovery times of 5 and 4 s, respectively [39]. Meanwhile, Ti_3_C_2_ MXene-based sensors demonstrated lower responses towards ethanol (0.7–5.4%), NO_2_ (1.2–5.4%), CO_2_ (1.5%), H_2_ (3.7%), IPA (0.53%), and NO (2.4%) compared to NH_3_ response behavior. These results confirm the excellent selectivity and durability of Ti_3_C_2_ MXene-based composites for reliable NH_3_ detection at room temperature. These advancements in the NH_3_ gas-sensing abilities of Ti_3_C_2_ MXene-based composites are summarized in Figure 8.

## 6. Conclusions

The incorporation of Ti_3_C_2_T_x_ MXene into various nanostructured interfaces has successfully contributed to room-temperature NH_3_ and H_2_ gas-sensing applications. The intrinsic properties of Ti_3_C_2_T_x_ MXene, including high electrical conductivity (facilitating charge transfer), surface termination groups (promoting hydrogen bonding and molecular interaction), and layered architecture (enhancing gas diffusion), play crucial roles in improving sensing performance at room temperature. The structural and chemical features have enabled development of robust gas sensors with high sensitivity ranging from ppm to ppt concentrations and ultrafast response characteristics. As a proof of concept, Ti_3_C_2_ MXene@sulfur nanosheets achieved exceptional response/recovery times of 5/4 s under ppt-level of NH_3_ [39]. Moreover, the Ti_3_C_2_ MXene-based composites maintained stable sensing performance under humid environments and exhibited long-term stability exceeding 50 days. For practical H_2_ leak detection, the integration of Ti_3_C_2_ MXene-based sensors with an alarm circuit and LED indicator was successfully demonstrated, where the LED was activated upon exposure to 4% H_2_ gas [21]. Additionally, flexible Ti_3_C_2_T_x_ MXene/graphene hybrid fibers woven into laboratory coats have demonstrated the potential for wearable room-temperature NH_3_ sensing applications [40]. Overall, the synergistic coupling of 2D Ti_3_C_2_ MXene with diverse semiconductor nanostructures has significantly enhanced the selectivity and sensing capability of H_2_ and NH_3_ gas sensors at room temperature.

## 7. Future Perspectives

Although Ti_3_C_2_T_x_ MXene-based composites have demonstrated outstanding room-temperature H_2_ and NH_3_-sensing performance, the following key strategies can further strengthen their H_2_ and NH_3_-sensing capabilities.

1. The anchoring of isolated single metal atoms onto Ti_3_C_2_ MXene surfaces can accelerate gas molecule dissociation and activation through tunable adsorption energies. Highly dispersed metallic centers act as adsorption intermediates, which modify electrical properties through enhanced electron transfer and improved sensitivity.

2. Controlled engineering of selective surface termination groups can promote selective gas adsorption and facilitate charge transfer between gas molecules and sensing materials to improve sensing performance.

3. Advanced in situ characterization techniques, including in-situ X-ray absorption spectroscopy, Raman spectroscopy, and FTIR are essential for understanding gas adsorption mechanisms and identifying intermediate species formed during room-temperature gas sensing.

4. Theoretical investigations into Ti_3_C_2_ MXene-based H_2_ and NH_3_ gas sensors remain relatively limited. Therefore, comprehensive DFT studies are required in order to understand the adsorption behavior of gas molecules on different surface terminations, including –OH, –O, and –F-functionalized Ti_3_C_2_ MXenes.

5. Further modification of the Ti_3_C_2_ MXene structure (into a monolayer or few layers) can enable ultrafast response and recovery kinetics for ppb-level detection through improved gas adsorption, charge trapping, and charge transport mechanisms. Also, intercalation of various nanostructures between Ti_3_C_2_ MXene nanosheets expands the interlayer spacing and increases the accessible surface area, thereby enhancing the gas-sensing performance.

Overall, Ti_3_C_2_ MXene-based composites provide a promising platform for advancing room-temperature H_2_ and NH_3_ gas sensing from ppm to ppb detection regimes.

## Figures and Tables

**Table 1:** Room-temperature H_2_ gas-sensing ability of Ti_3_C_2_ MXene-based nanostructures.

Composite	Working Temperature	Response/ Recovery Time (s)	Response (%)	Gas Concentration /LOD (ppm)	Gas Sensing Equation	Stability (Cycles)/Long-Term Stability (Days)	Reference
*Ti_3_C_2_-MXene based composites for H_2_ sensing*							
*Ti_3_C_2_/Pd nanostructures*							
Ti_3_C_2_@Pd CNC	Room temperature	32 ± 7/161 ± 23 s	23.0 ± 4.0 (Sensitivity)	4% H_2_	(I_g_ − I_0_)/I_0_ × 100	8/—	[21]
Pd-Ti_3_C_2_T_x_	Room temperature	—	56	100	|R_g_ − R_a_|/R_a_ × 100	5/90 at 50 ppm	[22]
Pd-NTO NRs	Room temperature	1.1/13.5	12	1% H_2_/0.8	(I_gas_ − I_air_)/I_air_	11 at 5000 ppm/8	[23]
Pd=Pt/Ti_3_C_2_T_x_	Room temperature	1/—	24.6	200/1	|R_a_ − R_g_|/R_a_ × 100	10/60 at 200 ppm	[24]
*Ti_3_C_2_ MXene derived C/TiO_2_*							
Ti_3_C_2_ MXene derived C/TiO_2_	Room temperature	13/42	62.2	40,000/50	|R_a_ − R_g_|/R_a_ × 100	—/30 at 300 °C	[26]
*Ti_3_C_2_ MXene/organic frameworks*							
DAAQ-COF/with Ti_3_C_2_	Room temperature	29/18	—	1 ppm/—	—	5/25	[27]
*Ion beam irradiated Ti_3_C_2_ MXene*							
N^+^ ion-beam induced Ti_3_C_2_ MXene	Room temperature	98/109	2.1	3000	R_o_/R_g_	10/10	[30]

**Table 2:** Room-temperature NH_3_ gas-sensing ability of Ti_3_C_2_ MXene-based nanostructures.

Composite	Working Temperature	Response/ Recovery Time (s)	Response (%)	Gas Concentration /LOD (ppm)	Gas Sensing Equation	Stability (Cycles)/Long-Term Stability (Days)	Reference
*Ti_3_C_2_ MXene based composites for NH_3_ sensing*							
*Pure Ti_3_C_2_-MXene*							
Ti_3_C_2_T_x_	Room temperature	—	1.22	1/—	|R_g_ − R_a_|/R_a_ × 100	5 at 100 ppm	[31]
Ti_3_C_2_T_x_/Ti_3_AlC_2_	Room temperature	12/60	11	200/50 ppb	|R_g_ − R_a_|/R_a_ × 100	—/120	[32]
Ti_3_C_2_T_x_	Room temperature	—	0.67	100/—	ΔR/R_a_ × 100	6/—	[33]
*Metal ions intercalated Ti_3_C_2_ MXene*							
K^+^, Na^+^, Ca^2+^ and Mg^2+^ cations intercalated Ti_3_C_2_T_x_ MXene	Room temperature	—	0.36	100/10	ΔR/R_0_ × 100	—	[34]
*Alkalized Ti_3_C_2_ MXene*							
NaOH alkalized Ti_3_C_2_T_x_	Room temperature	73/38 (200 ppm)	100.3	500/5	|R_g_ − R_a_|/R_a_ × 100	5/17	[36]
KOH alkalized Ti_3_C_2_T_x_	Room temperature	170/— at 100 ppm	35.7	200/	—	8/50	[37]
*Partially alkalized Ti_3_C_2_ MXene*							
Partially oxidized Ti_3_C_2_T_x_	Room temperature	67/157	21.3	10/—	ΔR/R_g_	4/21 at 500 ppm	[38]
*Ti_3_C_2_ MXene/*sulfur							
Ti_3_C_2_@Sulfur	Room temperature	5/4	375	3 ppt/—	—	5/20	[39]
*Ti_3_C_2_ MXene/Graphene*							
Ti_3_C_2_T_x_ MXene/graphene	Room temperature	—	7.21	100/10	ΔR/R_0_	2000 (bending)	[40]
Ti_3_C_2_T_x_/graphene	Room temperature	26/148	25.5	100/0.5	r_m_ − r_g_/r_g_ × 100	3/16	[41]
*Ti_3_C_2_ MXene/semiconductors*							
Ti_3_C_2_T_x_/SnO_2_	Room temperature	36/44	40 at 46% RH	50/4.29 ppb	|R_a_ − R_g_|/R_a_ × 100	5 at 30 ppm/60 at 50 ppm	[42]
Ti_3_C_2_T_x_/SnO	Room temperature	61/119	7.8	200/1	R_a_/R_g_	4/22	[43]
In_2_O_3_/Ti_3_C_2_T_x_	Room temperature	3/2	60.6	5/—	|R_a_ − R_g_|/R_a_ × 100	3/30	[44]
NiO/Ti_3_C_2_T_x_	Room temperature	60/19	6.13	50/10	|R_g_ − R_a_|/R_a_ × 100	15/15	[46]
ZnO@Ti_3_C_2_T_x_	Room temperature	92/104	39.16	20/89.1 ppb	|R_a_ − R_g_|/R_a_ × 100	10/—	[47]
Ti_3_C_2_T_x_/MoS_2_/PPy	Room temperature	43.6/40.8	23.1	10/—	|R_g_ − R_a_|/R_a_ × 100	5/7	[48]
Bi_2_O_3_/Ti_3_C_2_T_x_	Room temperature	61/164	61	100/5	|R_g_ − R_a_|/R_a_ × 100	6/20	[49]
Ti_3_C_2_T_x_/GO/CuO/ZnO	Room temperature	26/25	96.3	200/4.1	|R_a_ − R_g_|/R_a_ × 100	6/45	[50]
*Ti_3_C_2_ MXene/Ag and Au metals*							
Ag-loaded Ti_3_C_2_T_x_	Room temperature	511/605	4.96	10/0.5	|R_g_ − R_a_|/R_a_ × 100	—/30	[51]
Au/Ti_3_C_2_T_x_	Room temperature	87/217	16	100/—	|R_g_ − R_a_|/R_a_ × 100	6/—	[52]
*Ti_3_C_2_ MXene/polymers*							
PEDOT:PSS/nitrogen doped Ti_3_C_2_T_x_	Room temperature	280/393.6	13 at 36% RH	10/—	|R_g_ − R_0_|/R_0_ × 100	4/17	[53]
PVDF-ZIF-67/Ti_3_C_2_T_x_	Room temperature	37.8/34.5	10.56	5/(9 ppt and 1 ppb)	|R_g_ − R_a_|/R_a_ × 100	5/35	[54]
PANI/Ti_3_C_2_T_x_	Room temperature	—	55.90	20/5	|R_g_ − R_a_|/R_a_ × 100	6/21	[55]
*Ti_3_C_2_ MXene/metal hydroxides*							
Ni(OH)_2_/Ti_3_C_2_T_x_	Room temperature	78/108 s	13.69	80/1	|Rg − Ra|/R_a_ × 100	5/30 at 50 ppm	[56]
Co(OH)_2_/Ti_3_C_2_T_x_	Room temperature	39/49	14.7	100/5	|R_a_ − R_g_|/R_a_ × 100	5/30	[57]
*Ti_3_C_2_ MXene/MOFs*							
Ni_3_(HITP)_2_/Ti_3_C_2_	Room temperature	9/13	33	30/0.1	|R − R_0_|/R_0_	5/50	[58]
*Ti_3_C_2_ MXene/single atoms*							
ME-Ni–N–C/Ti_3_C_2_T_x_	Room temperature	30/80 s	33.2	5/27.0 ppb	|R_a_ − R_g_|/R_a_ × 100	5/24	[59]
*Ti_3_C_2_ MXene/*cationic polyacrylamide							
CPAM/Ti_3_C_2_T_x_	Room temperature	12.7/14.6	3.1	150	(I_g_ − I_0_)/I_0_	2 at 200 ppm/10	[60]

## Data Availability

Data will be made available on request.
